# Sensitivity to splicing modulation of *BCL2* family genes defines cancer therapeutic strategies for splicing modulators

**DOI:** 10.1038/s41467-018-08150-5

**Published:** 2019-01-11

**Authors:** Daniel Aird, Teng Teng, Chia-Ling Huang, Ermira Pazolli, Deepti Banka, Kahlin Cheung-Ong, Cheryl Eifert, Craig Furman, Zhenhua Jeremy Wu, Michael Seiler, Silvia Buonamici, Peter Fekkes, Craig Karr, James Palacino, Eunice Park, Peter G. Smith, Lihua Yu, Yoshiharu Mizui, Markus Warmuth, Agustin Chicas, Laura Corson, Ping Zhu

**Affiliations:** H3 Biomedicine, Inc., 300 Technology Square, Cambridge, MA 02139 USA

## Abstract

Dysregulation of RNA splicing by spliceosome mutations or in cancer genes is increasingly recognized as a hallmark of cancer. Small molecule splicing modulators have been introduced into clinical trials to treat solid tumors or leukemia bearing recurrent spliceosome mutations. Nevertheless, further investigation of the molecular mechanisms that may enlighten therapeutic strategies for splicing modulators is highly desired. Here, using unbiased functional approaches, we report that the sensitivity to splicing modulation of the anti-apoptotic *BCL2* family genes is a key mechanism underlying preferential cytotoxicity induced by the SF3b-targeting splicing modulator E7107. While *BCL2A1*, *BCL2L2* and *MCL1* are prone to splicing perturbation, *BCL2L1* exhibits resistance to E7107-induced splicing modulation. Consequently, E7107 selectively induces apoptosis in BCL2A1-dependent melanoma cells and MCL1-dependent NSCLC cells. Furthermore, combination of BCLxL (*BCL2L1*-encoded) inhibitors and E7107 remarkably enhances cytotoxicity in cancer cells. These findings inform mechanism-based approaches to the future clinical development of splicing modulators in cancer treatment.

## Abstract (web summary)

Small molecule modulators of RNA splicing have therapeutic potential in tumours bearing spliceosome mutations. Here, the authors identify *BCL2* genes have differential sensitivities to SF3b-targeting splicing modulators and combination of SF3b-targeting splicing modulators and BCLxL inhibition induces synergistic cytotoxicity in cancer cells.

## Introduction

Discovering cancer-relevant molecular mechanisms and biomarkers to inform therapeutic strategies for cancer patients is a central focus in precision oncology^1,2^. This approach has been successfully applied in targeted therapy, where preclinical research and clinical studies have offered mechanistic insights to empower cancer treatment^3^. Despite recent advances in genomic, immunological, and functional understandings of cancer, the promise of single-agent targeted cancer therapy remains unfulfilled. Combination therapies are believed to enhance the curative potential for the majority of cancers. Preclinical and clinical evidence indicates that combination therapies can help to overcome incomplete response and therapeutic resistance of single-agent treatment of cancer^1,4,5^. Nevertheless, development of efficacious combination therapies has been highly challenging, in part due to the lack of efficient preclinical approaches that are predictive of clinical combination activity. Mechanism-based drug combination strategies have been developed by taking advantage of target/pathway-related biological findings from basic research. There are emerging translational successes in clinical applications of combination of targeted therapies, exemplified by the Food and Drug Administration (FDA)-approved combination of mitogen-activated protein kinase pathway inhibitors vemurafenib (RAF inhibitor) and cobimetinib (mitogen-activated extracellular signal-regulated kinase kinase inhibitor) for treating melanoma patients bearing *BRAF* mutations^6,7^. However, given the complexity of mechanisms of action, it has been particularly strenuous to identify single agent or combination therapeutic strategies for a broad range of anticancer agents, particularly those targeting the essential cellular pathways.

Modulation of RNA splicing by small molecules represents a new therapeutic approach for myeloid malignancies and solid tumors bearing splicing gene mutations, e.g., recurrent mutations in *SF3B1*, *SRSF2*, and *U2AF1*^8–10^. The spliceosome “sickness” associated with these mutations is believed to be the “Achilles’ heel” of these malignancies, offering therapeutic opportunities to treat these cancers with hypersensitivity to the splicing modulators^11–13^. Recently, a pladienolide derivative H3B-8800 was developed as an oral splicing modulator that preferential kills spliceosome-mutant cancer cells in preclinical models and is currently being tested in phase 1 clinical trials^13^. In addition to spliceosome mutations in cancer, it is of great interest to explore other molecular mechanisms conferring and/or biomarkers associated with hypersensitivity to splicing modulators. Recently, oncogenic activation of MYC has been shown to impact RNA splicing fidelity by upregulating the small nuclear ribonucleoprotein particles, including PRMT5^14,15^. Intriguingly, RNAi screening and pharmacological inhibition further indicate a synthetic lethality relationship between MYC activation and core spliceosome inhibition, e.g., perturbation of the SF3b complex subunits PHF5A and SF3B1^16–18^. SF3b-targeting splicing modulators have also been employed in disruption of hard-to-drug oncogenes, e.g., *MCL1*, through mis-splicing as an alternative approach to targeting these oncogenes required for tumor survival^19–21^. These observations suggest the broad potential of small molecule splicing modulators as a new approach for cancer treatment.

Here, using unbiased pooled short hairpin RNA (shRNA) screening, drug sensitivity profiling in a large cancer cell line panel, and whole-transcriptome sequencing (RNA-seq), we reveal that the differential sensitivity to splicing modulation of BH domain containing antiapoptotic *BCL2* family genes provides mechanism-based therapeutic strategies for SF3b-targeting small molecule splicing modulator. We use the small molecule splicing modulator E7107 to show that knockdown of *BCL2L1* sensitizes its cell-killing activity, while high expression of *BCL2L1* is associated with decreased cytotoxicity induced by E7107. In contrast, endogenous amplification/high expression of *MCL1* or *BCL2A1*, which confers “oncogene addiction” in tumor cells, is associated with hypersensitivity to E7107-induced cell death. Mechanistically, *MCL1* and *BCL2A1* transcripts are sensitive, whereas *BCL2L1* is more resistant to splicing perturbation. We further validate that splicing modulator induces selective apoptosis in cancer cell lines with endogenous amplification and high expression of *MCL1* or *BCL2A1*, and combination of splicing inhibition and BCLxL (encoded by *BCL2L1*) inhibition induces a synergistic cytotoxicity in cancer cells. Taken together, we propose therapeutic strategies for small molecule splicing modulators based on a molecular mechanism involving differential sensitivity to splicing modulation and dependency on antiapoptotic genes in cancer cells.

## Results

### Loss of *BCL2L1* sensitizes splicing modulator E7107

**Fig. 1:**
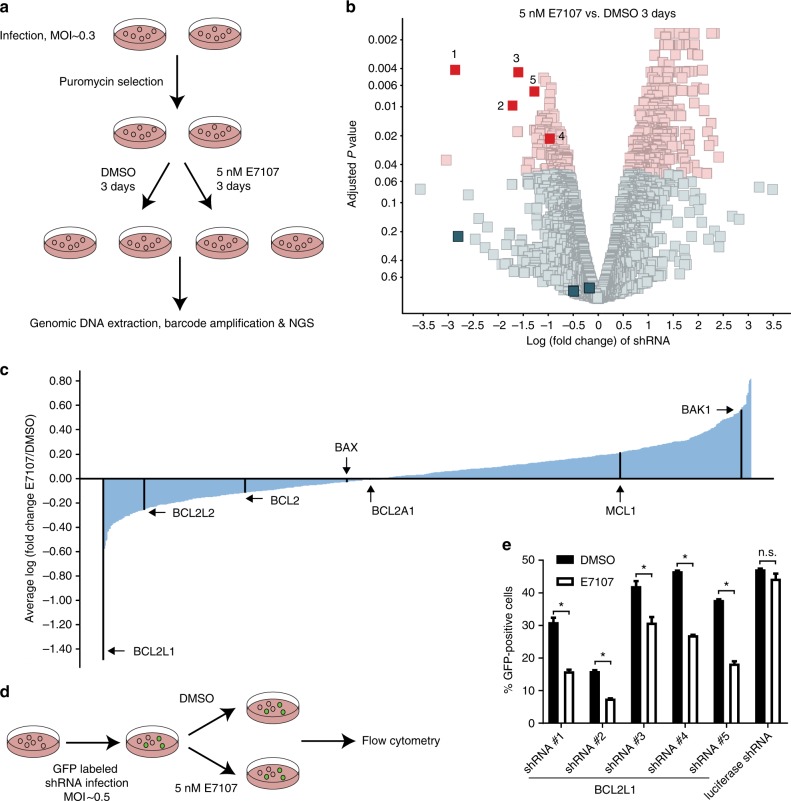
Pooled shRNA screen identifies *BCL2L1* as a sensitizing gene for splicing modulator E7107. **a** Schematic representation of the pooled shRNA screening in NALM6 cells treated with solvent DMSO or E7107. **b** Volcano plot demonstrating the log_2_(fold change) and adjusted *p* value (moderated *t* test by limma) of each shRNA in the pool screen (E7107 vs. DMSO, biological duplicates). For log_2_(fold change), negative and positive numbers represent drop-out (sensitization) and enrichment (resistance) phenotype, respectively, in combination with E7107 treatment. Red dots show shRNAs that are significantly (adjusted *p* < 0.05) altered in the screen. **c** Waterfall plot showing the average log_2_(fold change) (E7107 vs. DMSO) of shRNAs targeting the same gene included in the shRNA pool. Five antiapoptotic and two proapoptotic *BCL2* family genes were marked in black. **d** Schematic representation of a GFP-tracking phenotypic validation using single shRNAs against *BCL2L1* in NALM6 cells. **e** FACS analysis of the percentage of GFP-positive NALM6 cells infected by individual shRNAs after 72 h treatment with DMSO or E7107. Data represent means ± SD of biological duplicates. *p < 0.05, n.s. not significant, *p* > 0.05 by Student’s *t* test

To search for potential sensitizing targets and illustrate mechanism of action of splicing modulators, we carried out shRNA screens in NALM6 B cell acute lymphoblastic leukemia cells in the absence or presence of the SF3b-targeting splicing modulator E7107, a pladienolide derivative^17,18,22^. Specifically, NALM6 cells were infected with a pooled shRNA library containing 6500 individually barcoded hairpins targeting 841 different genes (~8 shRNAs per gene) covering a broad range of cellular processes associated with splicing, apoptosis, epigenetics, and signaling transduction that show high actionability for drug discovery (Supplementary Data 1). After puromycin selection of the infected cells, each replicate of infected NALM6 cells were split equally and treated with either dimethyl sulfoxide (DMSO) or 5 nM E7107 for 3 days (~GI_90_, the concentration that causes 90% growth inhibition) before sample collection. Unique barcodes from each shRNA vector were recovered from extracted genomic DNAs and subjected to next-generation sequencing (NGS) (Fig. 1a). To uncover sensitizing candidate targets for E7107, we compared the normalized read counts of each barcoded shRNA in E7107-treated samples to those of the DMSO-treated samples (Fig. 1b and Supplementary Data 2). Strikingly, five out of the eight shRNAs against *B Cell CLL/Lymphoma 2 like 1* (*BCL2L1*) that encodes the pro-survival protein BCLxL showed significant (adjusted *p* < 0.05 by moderated *t* test in R limma package) reduction upon E7107 treatment in comparison to DMSO controls (Fig. 1b and Supplementary Data 2). Consistent with the phenotypes of individual shRNA, gene-level analysis of the average fold changes elicited by individual shRNAs targeting the same gene showed that knockdown of *BCL2L1* induced the most robust depletion/sensitization in E7107-treated samples among 841 genes included in the pooled shRNA screens (Fig. 1c). In contrast, shRNAs against other BH domain-containing antiapoptotic genes (*BCL2*, *BCL2A1*, *BCL2L2*, and *MCL1*) or proapoptotic genes (*BAK1* and *BAX*) did not show remarkable sensitizing phenotypes upon E7107 treatment (Fig. 1c). Indeed, *BAK1* shRNAs showed a trend of desensitizing E7107, consistent with its role in proapoptosis (Fig. 1c). We also validated that NALM6 cells expressed most of the BH domain-containing *BCL2* family genes (Supplementary Fig. 1). To further validate the effect of these five positive shRNA hits against *BCL2L1*, each shRNA was cloned into a green fluorescent protein (GFP)-expressing vector. Each viral shRNA was introduced into NALM6 cells to achieve about 30–50% infection rate as measured by the percentage of GFP-positive cells. Therefore, we were able to follow the survival of both GFP-positive *BCL2L1*-depleted cells and GFP-negative uninfected control cells within the same well under identical treatment conditions (Fig. 1d). After 3 days of treatments, GFP-positive cells showed significant reduction (*p* < 0.05 by Student’s *t* test) in the presence of 5 nM E7107 in comparison to DMSO treatments, whereas the negative control shRNA targeting luciferase did not sensitize NALM6 cells to splicing modulator treatment (Fig. 1e). These individual shRNA data confirmed the pooled shRNA screen results, indicating that *BCL2L1* acts as a resistant mechanism for E7107 and can function as a sensitizing target for splicing modulator treatment.

### *BCL2L1* is a marker of insensitivity to splicing modulators

**Fig. 2:**
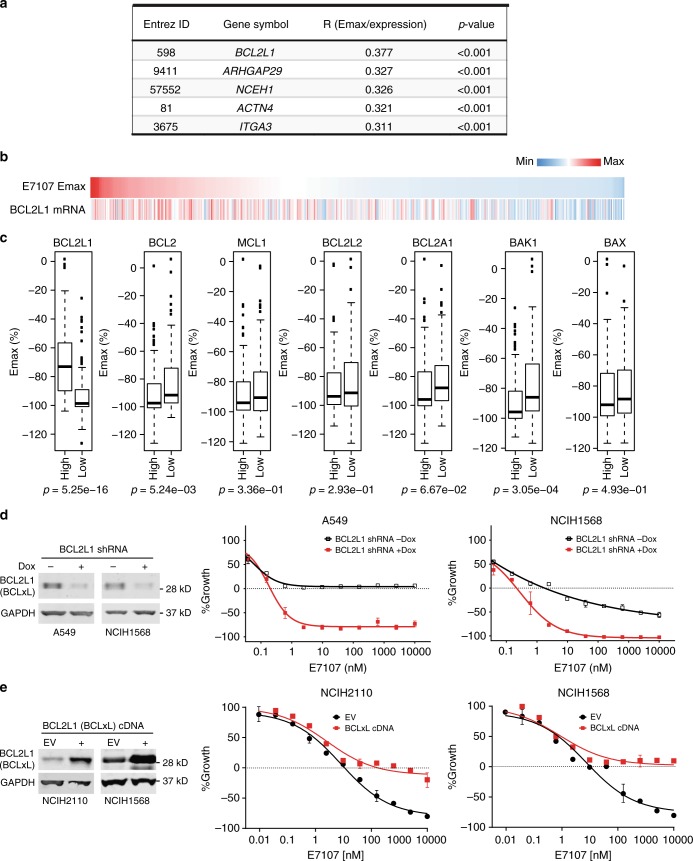
Cell line panel screen of drug sensitivity identifies *BCL2L1* expression as a marker of insensitivity to E7107. **a** Top five genes whose mRNA expression positively correlated with maximum effect of cell killing (Emax) of E7107 profiled in 478 cancer cell lines. Pearson’s correlation coefficient *R* and *p* values were calculated using R package Hmisc 4.1-0. Lower Emax value indicates more robust cell killing activity. **b** Heatmap demonstrating the positive correlation between Emax of E7107 and *BCL2L1* mRNA expression in 478 cancer cell lines. The heatmap was sorted by Emax of E7107 in cancer cell lines from high (less sensitive) to low (more sensitive). **c** Box plots showing the distribution of Emax (%) of E7107 in two groups of profiled cell lines defined by expression levels of each *BCL2* family genes. For each *BCL2* family genes, cell lines with expression level higher than the third quartile are classified into “high” and cell lines with expression level lower than the first quartile are classified into “low.” *p* Values were calculated using Student’s *t* test. **d** Growth-inhibitory activity of E7107 in lung cancer cell lines A549 and NCIH1568 upon doxycycline-induced shRNA knockdown of *BCL2L1*. Left, Western blot analysis of BCLxL(encoded by *BCL2L1*) knockdown; Right, Growth curves of two cell lines measured by CellTiter-Glo. Data represent means ± SD of biological triplicates. **e** Growth-inhibitory activity of E7107 in lung cancer cell lines NCIH2110 and NCIH1568 upon stable cDNA expression of *BCL2L1* (BCLxL). Left, Western blot analysis of BCLxL(encoded by *BCL2L1*) overexpression; Right, Growth curves of two cell lines measured by CellTiter-Glo. Data represent means ± SD of biological triplicates

In order to interrogate whether the functional connection between splicing modulation and *BCL2L1* exists generally across different cancer cells/lineages, we performed drug sensitivity assays for E7107 (9 concentrations ranging from 0.15 nM–10 μM) in 478 genetically characterized human cancer cell lines covering a variety of different hematological malignancies and solid tumor types (Supplementary Data 3). We conducted an unbiased analysis of correlation between the maximum killing effect (Emax) of E7107 and individual gene expression in these cell lines. Remarkably, *BCL2L1* is ranked as the top gene whose expression levels correlated with Emax values of E7107 in the panel of 478 cell lines, indicating that cell lines with high expression levels of *BCL2L1* are less sensitive to E7107-induced cell death (Fig. 2a, b). To our knowledge, none of the other top hits (Fig. 2a) has been clearly shown to be splicing or apoptosis related. Furthermore, *BCL2L1* is the only overlap gene among the top hits of both pooled shRNA screen (Supplementary Data 2) and cell line drug sensitivity screen (Supplementary Data 3). We further investigated the correlation in specific tissue types/lineages, demonstrating that *BCL2L1* remains one of the top-ranked genes whose expression is associated with Emax of E7107 in leukemia, lung, and ovarian cancer cell lines (Supplementary Fig. 2a). In contrast, other antiapoptotic *BCL2* family genes, e.g., *MCL1*, *BCL2*, *BCL2L2*, and *BCL2A1*, or proapoptotic genes *BAK1* and *BAX* did not show this trend of positive correlation (Fig. 2c). The association appeared to be selective for SF3b-targeting splicing modulators, because *BCL2L1* expression was also the top positive correlation marker with the Emax of a structurally different SF3b modulator herboxidiene^23^ (Supplementary Fig. 2b). In contrast, *BCL2L1* expression was not correlated with the sensitivity to the SRPK-inhibiting splicing modulator SRPIN340^24^, the RBM39/DCAF15-targeting splicing modulator tasisulam^25,26^ (Supplementary Fig. 2c), or the pan-cytotoxic agent proteasome inhibitor bortezomib in the cell line panel (Supplementary Fig. 2d). These results are consistent with the finding that knockdown of *BCL2L1* sensitized NALM6 cells to E7107, further strengthening the role of *BCL2L1* expression in modulation of E7107-induced cytotoxicity. To validate whether the expression level of *BCL2L1* is causative for insensitivity to E7107 in cells, we knocked down *BCL2L1* in A549 and NCIH1568 lung cancer cells using inducible shRNA targeting independent *BCL2L1* sequences different from the shRNAs used in the pooled screening (Fig. 1e). Reduction of *BCL2L1* converted splicing modulator E7107 from cytostatic to cytotoxic in A549 cells and induced more robust cytotoxicity in NCIH1568 cells upon E7107 treatment (Emax from about −50% to −100%) (Fig. 2d). Moreover, overexpression of *BCL2L1* (encodes BCLxL) cDNA antagonized splicing modulator-induced cytotoxicity in two cell lines (Fig. 2e). Together, these data indicate that low *BCL2L1* expression is an intrinsic determinant of cytotoxicity induced by splicing modulators.

### Differential sensitivity of *BCL2* genes to splicing modulation

We next sought to explore the underlying mechanisms of the synergistic effect of combining BCLxL (encoded by *BCL2L1*) inhibition with splicing modulator E7107, which may assist to shed light on the mechanism-based therapeutic strategies of splicing modulators in cancer treatment. It has been shown that the homeostasis of apoptosis-related proteins in cells determines the state of viability or programmed cell death^27,28^. Given the perturbation/disruption nature of splicing modulation, we focused on the antiapoptotic *BCL2* family genes, which encode BH domain-containing pro-survival proteins playing a particularly important role in regulating apoptosis^29,30^. Interestingly, the RNA levels of *BCL2* family genes exhibit a differential expression pattern in cancer cell lines and tumor tissues from The Cancer Genome Atlas (TCGA). While *MCL1*, *BCL2L1*, or *BCL2L2* were ubiquitously expressed, *BCL2* and *BCL2A1* demonstrated tissue/lineage-selective expression with very low/no expression across most of the cancer cell lines and TCGA tumor tissues (Supplementary Fig. 3a, b). We hypothesized that the expression levels and/or sensitivity to splicing modulators of these genes might be decisive factors for cytotoxicity induced by splicing modulator E7107.

**Fig. 3:**
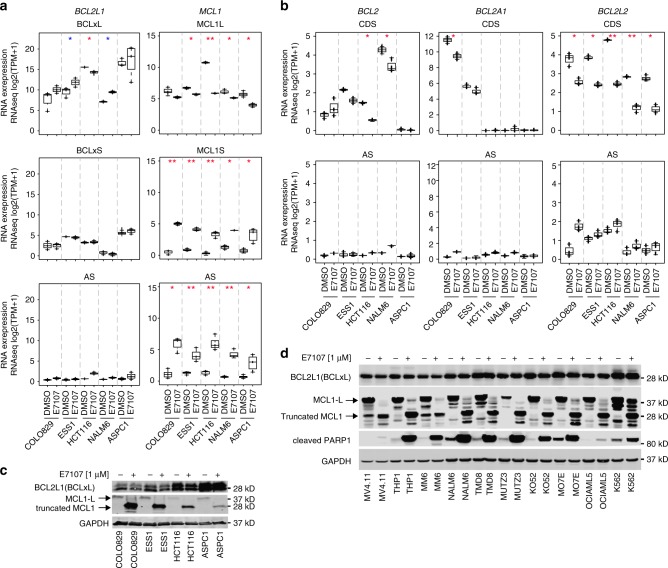
Antiapoptotic *BCL2* family genes exhibit differential sensitivity to E7107-induced splicing modulation. **a** RNA-seq analysis of *BCL2L1* and *MCL1* upon E7107 treatment (at GI50) in cancer cell lines COLO829 (35 nM for 6 h), ESS1 (16 nM for 6 h), HCT116 (50 nM for 6 h), NALM6 (15 nM for 6 h), and ASPC1 (41 nM for 6 h). RNA expression levels represent RNA reads for corresponding coding sequence (CDS) of the functional protein isoforms (L antiapoptotic long form, S proapoptotic short form) or aberrant splicing (AS) events that are not predicted to encode functional proteins. **b** RNA-seq analysis of *BCL2*, *BCL2A1*, and *BCL2L2* under same conditions shown in **a**. *p < 0.01, **p < 0.001; red asterisks: proapoptotic events with median expression log_2_(TPM + 1) > 2, blue asterisks: antiapoptotic events with median expression log_2_(TPM + 1) > 2. **c** Western blot analysis of BCLxL and MCL1 protein expression upon treatment with E7107 (1 μM for 6 h). GAPDH was used as the loading control. **d** Western blot analysis of BCLxL, MCL1, and cleaved PARP1 protein expression in a panel of leukemia cell lines upon treatment with E7107 (1 μM for 6 h). GAPDH was used as the loading control

In order to examine the sensitivity to splicing modulation of *BCL2* family genes, we conducted whole-transcriptome RNA-seq for five cancer cell lines representing different lineages, including a melanoma cell line COLO829, an endometrial cancer cell line ESS1, a colorectal cancer cell line HCT116, a pancreatic adenocarcinoma cell line ASPC1, and NALM6. Given the fact that alternative and/or aberrant splicing (AS) events are not well characterized for all genes or junctions, we investigated the mRNA reads covering the coding DNA sequences (CDS), which could represent the steady status of mRNA for translation to functional proteins, and the AS reads including intron retention and other mis-splicing events that would not be able to translate to any functional protein products (Supplementary Data 4). With regard to *MCL1* and *BCL2L1* genes, two CDS variants encoding the antiapoptotic long-form proteins (MCL1L and BCLxL) and the proapoptotic short-form proteins (MCL1S and BCLxS), respectively, were counted separately (Fig. 3a). Remarkably, five *BCL2* family genes exhibited differential sensitivity to splicing modulator E7107. Most of the *BCL2L1* transcripts, including the CDS encoding BCLxL or BCLxS and the AS transcripts, were not substantially regulated by E7107 in the cell lines tested (Fig. 3a, left panels). Of note, BCLxL transcripts are highly expressed and slightly induced by E7107 in some cell lines, whereas BCLxS or AS transcripts were expressed at low levels, suggesting that the key antiapoptotic function of BCLxL was not interfered (Fig. 3a, left panels). In contrast, *MCL1* transcripts were clearly regulated by E7107-triggered splicing modulation, with a reduction of the CDS transcripts encoding the antiapoptotic MCL1L protein, and a substantial induction of the AS transcripts and the alternatively spliced transcripts encoding the putative proapoptotic MCL1S protein (Fig. 3a, right panels). We also validated the RNA-seq data by reverse transcriptase quantitative polymerase chain reaction (RT-qPCR) using MCL1L and MCL1S-specific TaqMan probes in HCT116 cells (Supplementary Fig. 4a). Similarly, *BCL2L2* CDS transcripts were inhibited, whereas the AS transcripts were increased upon E7107 treatment in all cell lines (Fig. 3b, right panels). As lineage-selective *BCL2* family genes, both *BCL2* and *BCL2A1* showed a clear reduction of the CDS transcripts and induction of non-functional AS transcripts in cell lines expressing high levels of the corresponding genes (Fig. 3b, left and middle panels). To test whether the differential splicing regulation of *BCL2* family genes is SF3b modulator-selective, we again evaluated SRPIN340, tasisulam, and herboxidiene by RNA-seq. Unlike E7107, neither SRPIN340 nor tasisulam exhibited a clear splicing modulation of *MCL1* (Supplementary Fig. 4b). In contrast, a structurally different SF3b-targeting splicing modulator herboxidiene derivative is a potent regulator of the splicing of *MCL1* and *BCL2L2* genes but not *BCL2L1* (Supplementary Fig. 4c), suggesting that the splicing modulation on *BCL2* genes is similar between SF3b-targting molecules.

Finally, we confirmed that MCL1 full-length (MCL1L) protein was converted to a truncated short-form protein correlating with the results from RNA-seq, whereas BCLxL protein levels remained unaltered in response to E7107 treatment (Fig. 3c). In addition, we also evaluated the protein levels of BCLxL (encoded by *BCL2L1*) and MCL1 in 12 acute myeloid leukemia lines that express both proteins. Again, whereas BCLxL protein was not impacted by E7107, MCL1L protein was remarkably reduced and truncated short-form MCL1 proteins were produced by E7107 in all cell lines (Fig. 3d). Coincidentally, cleaved poly ADP-ribose polymerase 1 (PARP1), a sign of apoptosis, was also induced in all cell lines (Fig. 3d). In aggregate, these data indicate that expression pattern and sensitivity of the *BCL2* family genes to the SF3b-targeting splicing modulator E7107 are notably differential, which may contribute to the differential cytotoxicity induced by splicing modulator and inform the combination strategies for splicing modulator.

Based on the differential sensitivity of these antiapoptotic genes to splicing modulator E7107, and because of the cooperative role of *BCL2* family members in supporting cancer cell survival, we then pursued these therapeutic hypotheses: (1) E7107 may induce cytotoxicity preferentially in cancer cells with MCL1 or BCL2A1 dependency by splicing perturbation of these genes; and (2) E7107 may synergize with inhibitors of BCLxL (encoded by *BCL2L1*), whose splicing is insensitive to E7107, to potentiate the cytotoxicity in cancer cells.

### E7107 induces melanoma cell death by targeting *BCL2A1*

**Fig. 4:**
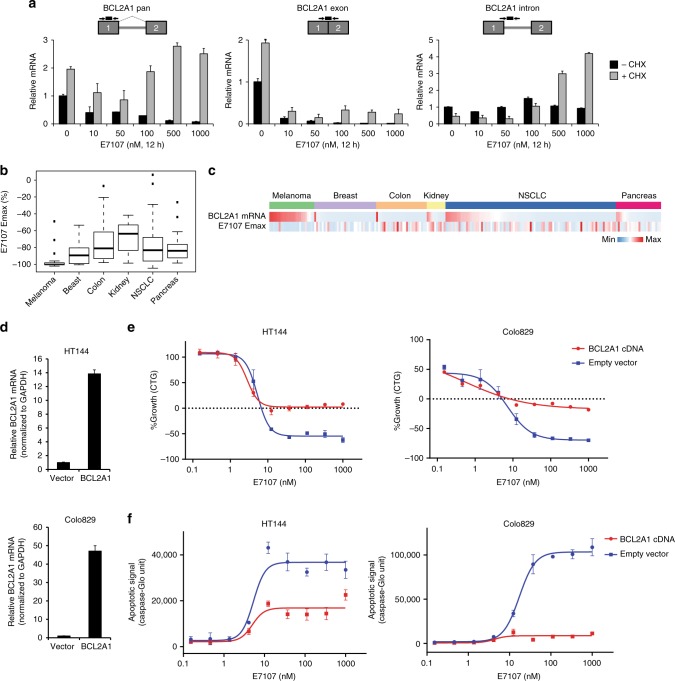
E7107 induces BCL2A1-dependent apoptosis in melanoma cell lines. **a** RT quantitative PCR (RT-qPCR) analysis of total mRNA levels (pan), exonic mRNA levels covering the junction of exons (exon), and pre-mRNA levels detecting the intron (intron) of *BCL2A1*. HT144 cells were pre-treated with 100 µg ml^−1^ cycloheximide (CHX) for 1 h, followed by addition of E7107 as indicated for 12 h to inhibit nonsense-mediated mRNA decay (NMD) before collecting RNA samples. Schematic representations of TaqMan primers and probes were shown. Boxes: exons; gray lines: introns; arrows: primers; and black lines: probes. **b** Box plots showing the distribution of E7107 Emax in the solid tumor cell lines separated by tissue/lineage: melanoma (*n* = 24), breast (*n* = 33), colon (*n* = 27), kidney (*n* = 10), lung (*n* = 91), and pancreas (*n* = 24). The box plots exhibit five number summary from bottom to top: minimum, first quartile, median, third quartile, and maximum/outliers. **c** Heatmap of *BCL2A1* mRNA expression (based on CCLE RNA-seq, sorted from high to low in the solid tumor cell lines separated by tissue/lineage) and E7107 Emax. **d** RT-qPCR analysis of *BCL2A1* mRNA levels (exon1/2 junction) in vector control (Vector) or stable *BCL2A1* cDNA-expressing (BCL2A1) melanoma cell lines. Data represent means ± SD of biological triplicates. **e** Growth curves measured by CellTiter-Glo (CTG) and **f** apoptosis induction curves measured by IncuCyte Caspase-3/7 Green Apoptosis Assay in melanoma cell lines stably transduced with vector control (empty vector) or *BCL2A1* cDNA (BCL2A1 cDNA) treated with E7107 for 72 h. Data represent means ± SD of biological triplicates

As an antiapoptotic *BCL2* family member and a target of the melanocytic transcription factor MITF, *BCL2A1* is highly and selectively expressed or amplified in melanoma^31^ (Supplementary Fig. 3). Moreover, it has been reported that BCL2A1 is a key pro-survival factor in melanoma cells^31^. As revealed by RNA-seq analysis (Fig. 3b), *BCL2A1* is downregulated by E7107. Therefore, we hypothesized a preferential cytotoxic activity of E7107 in melanoma cell lines. We first attempted to elucidate the splicing-relevant mechanism for *BCL2A1* reduction in melanoma cells. In HT144 cells treated with E7107, we observed a dose-dependent decrease of total *BCL2A1* mRNA expression, which is remarkably rescued by addition of a translational and nonsense-mediated mRNA decay (NMD) inhibitor cycloheximide, suggesting that canonical *BCL2A1* mRNA production is inhibited by splicing modulation through an NMD-dependent mechanism (Fig. 4a, left panel). This is further confirmed when we evaluated *BCL2A1* intron RNA, which represents the pre-mRNA, showing an accumulation by E7107 treatment in the presence of cycloheximide (Fig. 4a, right panel). In contrast, *BCL2A1* mature mRNA, measured by exon junction-specific detection, was decreased by E7107 regardless of the status of cycloheximide (Fig. 4a, middle panel). Together, these data indicate that splicing modulator E7107 inhibits *BCL2A1* expression through intron-retention-associated NMD in melanoma cells. Under the same treatment conditions, *BCL2L1* mRNA was not substantially altered by E7107 (Supplementary Fig. 5). Next we investigated the cytotoxic activity of E7107 in 24 melanoma cell lines. In general, melanoma cell lines, which all express relatively high level of *BCL2A1*, were extremely sensitive to E7107 treatment as measured by Emax (Fig. 4b). In comparison, other solid tumor cell lines of multiple tissue origins, including breast, colon, kidney, lung, and pancreas, demonstrated a diversity of sensitivity with only a minor subset showing high cytotoxicity to E7107 (Fig. 4b, c). This would suggest that splicing modulators, by inducing *BCL2A1* intron retention and subsequent NMD, may impinge on the BCL2A1 dependency to induce cell death in melanoma cells. In order to validate the functional role of BCL2A1, we expressed *BCL2A1* cDNA, which is resistant to splicing modulation, in HT144 and COLO829 cell lines (Fig. 4d). Convincingly, *BCL2A1* cDNA expression significantly compromised the cytotoxic effect of E7107 to cytostasis in both melanoma cell lines (Fig. 4e). Consistent with the cell viability effect, *BCL2A1* cDNA expression also strongly suppressed E7107-induced apoptosis (Fig. 4f). The BCL2A1-dependent cell death appears to be a specific mechanism for E7107, because *BCL2A1* cDNA was not able to rescue the cell death induced by cytotoxic agents, e.g., proteasome inhibitor bortezomib and pan-kinase inhibitor staurosporine (Supplementary Fig. 6). In aggregate, the data indicate that splicing inhibition of *BCL2A1* may trigger BCL2A1-dependent cytotoxicity, suggesting a potential therapeutic strategy for melanoma with high *BCL2A1* expression.

### E7107 induces cytotoxicity in MCL1-dependent cancer cells

**Fig. 5:**
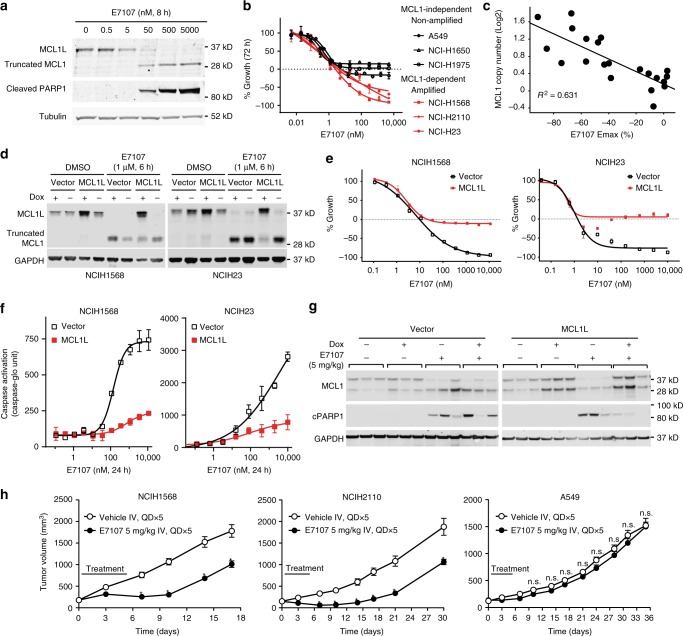
E7107 induces MCL1-dependent apoptosis in MCL1-overexpressed/dependent NSCLC cells. **a** Western blot analysis of MCL1 protein and cleaved PARP1 as an indicator as apoptosis. Tubulin was used as loading control. NCIH1568 cells were treated with E7107 as indicated. **b** Growth curves measured by CellTiter-Glo (CTG) in NSCLC cell lines. Data represent means ± SD of biological triplicates. **c** Correlation of *MCL1* copy number and E7107 Emax in 21 NSCLC cell lines. **d** Western blot analysis of MCL1 and GAPDH (loading control) protein expression in NSCLC cell lines transduced with vector control (Vector) or Dox-inducible expression of MCL1L cDNA (MCL1L). Cells were treated with DMSO or E7107 as indicated. **e** Growth curves measured by CellTiter-Glo (CTG); and **f** Caspase activation detected by Essen Biosciences IncuCyte Caspase-3/7 Green Apoptosis Assay. Data represent means ± SD of biological triplicates. NSCLC cell lines transduced with vector control (Vector) or Dox-inducible expression of MCL1L cDNA (MCL1L) in the presence of 300 ng ml^−1^ Dox. Cells were treated with E7107 as indicated. **g** Western blot analysis of NCIH1568 xenograft tumor samples. The engineered cell lines from **d** were used for the in vivo studies. **h** Tumor growth curves measured by tumor volume of three NSCLC xenograft models treated with vehicle or E7107. H1568: 8 mice/group; H2110: 6 mice/group; A549: 8 mice/group. Statistical significance was analyzed by the Holm–Sidak method in Prism. Each row was analyzed individually, without assuming a consistent SD. *p < 0.001; n.s. not significant, *p* > 0.1

*MCL1* is one of the most frequently amplified genes in cancer, including a subset of non-small cell lung carcinoma (NSCLC), which may confer dependence on high MCL1 levels for survival^32,33^. We have shown that MCL1 is one of the most sensitive *BCL2* family genes to splicing modulation induced by E7107 (Fig. 3a). Others have uncovered that splicing modulators meayamycin B, sudemycin, and spliceostatin A could modulate *MCL1* gene splicing, switching it from the MCL1L to MCL1S^19–21^. Therefore, we sought to examine whether E7107 could induce preferential lethality in MCL1-dependent NSCLC cells. We first identified a series of MCL1-amplified/high and MCL1-low NSCLC cell lines by western blot analysis (Supplementary Fig. 7a). Consistent with previous reports^33^, shRNA depletion of *MCL1* induced growth inhibition in MCL1-amplified/high cell lines but not MCL1-low cell lines while the shRNA effectively reduced MCL1 levels in all cell lines (Supplementary Fig. 7b, 7c). We next tested E7107 in NSCLC cells, confirming that *MCL1* gene is highly sensitive to E7107-induced splicing modulation, which led to downregulation of the pro-survival full-length (MCL1L) and induction of intron retention and a truncated short-form MCL1 (Fig. 5a). Furthermore, E7107 treatment induced cytotoxic effect on MCL1-amplified/high NSCLC cell lines but only cytostatic effect on MCL1-low cell lines, recapitulating the phenotypes of *MCL1* RNAi (Fig. 5b). We expanded the analysis to a panel of 22 NSCLC cell lines, which demonstrates that the Emax of E7107 showed negative correlation with *MCL1* copy numbers (*R*^2^ = 0.631) (Fig. 5c). This again indicates that high level of MCL1 confers better cytotoxicity to E7107 in NSCLC cells. Next, we re-expressed the MCL1L cDNA, which is resistant to E7107-induced splicing modulation, in two MCL1-dependent NSCLC cell lines by an inducible system, in order to evaluate whether it could rescue the MCL1-dependent cells from the cytotoxic effect of E7107 (Fig. 5d). Indeed, in MCL1-high/dependent H23 and H1568 cells, E7107-induced cytotoxicity was completely abolished by the expression of the MCL1L cDNA (Fig. 5e). Moreover, the apoptosis triggered by E7107 was clearly reduced by MCL1L cDNA as revealed by a caspase activity assay (Fig. 5f). The effect appeared to be selective to E7107 as MCL1L cDNA expression did not rescue cell death induced by the cytotoxic pan-kinase inhibitor staurosporine (Supplementary Fig. 8).

Next, we examined the activity of E7107 in xenograft NSCLC models. In the H1568 tumors engineered with inducible MCL1L cDNA, we observed that E7107 (5 mg kg^−1^ intravenous (i.v.) single dose) downregulated MCL1L mRNA (exons1/2/3) while MCL1S mRNA (exons1/3) and intron-retained isoforms were enhanced without doxycycline (Dox) in vivo (Supplementary Fig. 9). Moreover, E7107 reduced endogenous MCL1L protein levels and induced cleaved PARP1, an indicator of apoptosis, in the absence of Dox (Fig. 5g, right panel). Intriguingly, Dox-induced expression of exogenous MCL1L largely diminished the level of cleaved PARP1, suggesting that splicing disruption of the *MCL1* gene might be the major cause of E7107-induced cell death in vivo (Fig. 5g, right panel). In contrast, in tumors engineered with the control empty vector, E7107-mediated reduction of MCL1L protein levels and induction of cleaved PARP1 were consistent regardless of the Dox status (Fig. 5g, left panel). In order to test whether splicing inhibition of *MCL1* would selectively inhibit the MCL1-amplified/dependent NSCLC lines in vivo, we performed antitumor efficacy studies in three xenograft models: MCL1-dependent H1568 and H2110 and MCL1-independent A549. After the administration of E7107 (5 mg kg^−1^ i.v. q.d. for 5 consecutive days) in mice, both H1568 and H2110 tumor models showed a significant tumor growth inhibition, whereas A549 tumors were not inhibited significantly (Fig. 5h).

### Synergism of splicing modulators and BCLxL inhibitors

**Fig. 6:**
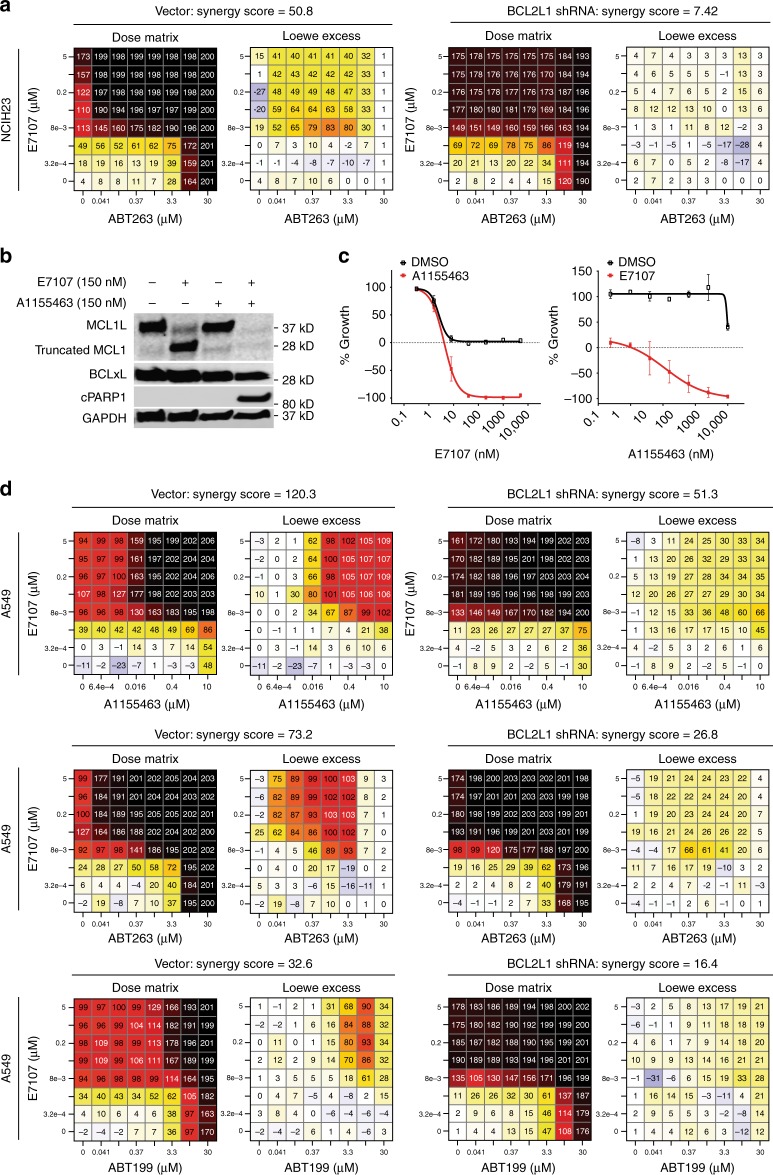
Combination of splicing modulator E7107 with BCLxL inhibitors induces synergistic cytotoxicity. **a** 8 × 8 dose matrix combination study for E7107 and ABT263 in MCL1-dependent NCIH23 cells stably transduced with vector control (Vector) or *BCL2L1* shRNA. Synergy scores were calculated by the Chalice software (Horizon Discovery). One representative of three independent experiments is shown. **b** Western blot analysis of MCL1, BCLxL, cleaved PARP1 (cPARP), and GAPDH (loading control) in MCL1-independent A549 cells treated with E7107 or BCLxL-specific inhibitor A1155463. **c** Growth curves measured by CellTiter-Glo (CTG) in A549 cells treated with single agent (black curves: left, E7107; right, A1155463) or in combination (red curves: left, E7107+40 nM A1155463; right, A1155463+40 nM E7107). Data represent means ± SD of biological triplicates. **d** 8 × 8 dose matrix combination study for E7107 and BCLxL/BCL2 inhibitors (A1155463, ABT263, and ABT199) in A549 cells stably transduced with vector control (Vector) or *BCL2L1* shRNA. Synergy scores were calculated by Chalice. One representative of three independent experiments is shown

Our functional screen data and the fact that E7107 can efficiently modulate *MCL1* and *BCL2A1* but is much less effective toward *BCL2L1* suggest that combination of E7107 with BCLxL inhibitors may provide enhanced cytotoxicity by broad targeting of key antiapoptotic *BCL2* family members. We first performed combination studies of E7107 and the BCL2/BCLxL inhibitor ABT263 (navitoclax, BCLxL Ki ≤ 0.5 nM)^34^ in MCL1-dependent NSCLC cell lines, showing that combination of two inhibitors led to synergistic cytotoxic effect in a 8 × 8 dose matrix with large excess over the Loewe additivity models (NCIH23 synergy score = 50.8, NCIH2110 synergy score = 50.2, NCIH1568 synergy score = 35.2; Fig. 6a and Supplementary Fig. 10, left panels). Importantly, shRNA depletion of BCLxL largely diminished this synergy between E7107 and ABT263, suggesting that the genetic shRNA perturbation and pharmacological inhibitor (ABT263) impinged on the same target BCLxL to achieve the synergistic effect (Fig. 6a and Supplementary Fig. 10, right panels).

**Fig. 7:**
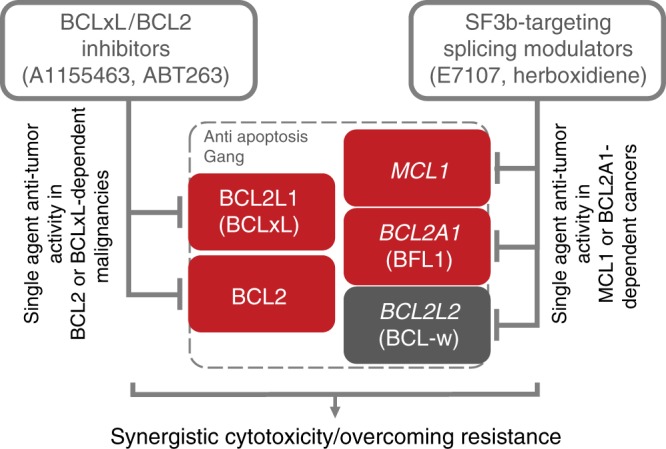
Mechanism of combinatory activity between SF3b-targeting splicing modulators and BCLxL/BCL2 inhibitors. SF3b-targeting splicing modulators preferentially perturb the RNA splicing of *MCL1* and *BCL2A1* but not *BCL2L1* (BCLxL), leading to selective cytotoxicity in MCL1- or BCL2A1-dependent cancer cells. In combination with BCLxL/BCL2 inhibitors, splicing modulators can enhance the cytotoxicity through a broader inhibition of the BCL2 family genes that act cooperatively in antiapoptosis/pro-survival in cancer cells. These findings inform mechanism-based approaches to the future clinical development of splicing modulators in cancer treatment. This combination strategy may offer effective repression of most of the cancer-relevant antiapoptotic BCL2 family members, thus broadening the impact of both compounds to a variety of indications, and potentially overcoming the resistance to the current BCL2/BCLxL-targeting agents. BCL2 family genes: red, confirmed oncogene-addiction role; gray: undetermined role

To investigate whether combination of splicing inhibitor and BCLxL inhibitor would induce cytotoxicity in MCL1-independent NSCLC cells, and to further confirm that BCLxL is the major mediator of synergistic activity, we used a BCLxL-specific inhibitor A1155463 (BCLxL Ki ≤ 0.01 nM)^35^ for combination studies in the MCL1 low A549 cell line that is cytostatic to E7107 (Figs. 2d and 5b). Here, although E7107 potently induced splicing modulation of MCL1 comparable to what we have observed in MCL1-dependent cells, neither E7107 nor A1155463 single-agent treatment resulted in PARP1 cleavage in A549 (Fig. 6b). Strikingly, combination treatment with E7107 and A1155463 led to a robust induction of cleaved PARP1, suggesting that cell death can only be triggered by combination (Fig. 6b). In line with the results, addition of 40 nM of A1155463 converted E7107 from a cytostatic (Emax = 0) to a potent cytotoxic agent (Emax = −100%) in A549 (Fig. 6c, left panel). Reciprocally, A1155463 demonstrated an LD50 (−50% lethality dose) at about 120 nM in the presence of 40 nM E7107, while single agent was completely inactive in the viability assays of A549 (Fig. 6c, right panel). We next conducted comprehensive 8 × 8 dose matrix combination assays in A549. Three BCLxL/BCL2 inhibitors (A1155463, ABT263 and ABT199)^34–36^ with differential selectivity and potency to BCL2 proteins were applied in combination with E7107. As the baseline control, single-agent “combination” of each compounds showed only additive activity in the Loewe model with synergy scores <5 (Supplementary Fig. 11). Convincingly, all three BCLxL/BCL2 inhibitor exhibited synergistic cytotoxicity with E7017 (Fig. 6d, left panels). Again, the synergism was largely dependent on BCLxL since knockdown of *BCL2L1* by shRNA substantially reduced the synergy scores (Fig. 6d, right panels). In addition, the synergism appeared to correlate with the potency of inhibition on BCLxL rather than on other BCL2 proteins (A1155463: Ki ≤ 0.01 nM, synergy score = 120.3; ABT263: Ki ≤ 0.5 nM, synergy score = 73.2; ABT199: Ki = 48 nM, synergy score = 32.6). In addition to E7107, we also observed a similar combinatory cytotoxicity between herboxidiene, a structurally different SF3b-targeting splicing modulator, and ABT263 in four NSCLC cell lines (Supplementary Fig. 12). Knockdown of BCL2L1 also largely diminished the synergistic activity (Supplementary Fig. 12), suggesting that it is a common phenomenon for some SF3b-targeting splicing modulators and BCLxL/BCL2 inhibitors. Taken together, these data are consistent with our previous observation from shRNA screen and demonstrate the potential of combining SF3b splicing inhibitors with BCLxL inhibitors to induce potent cancer cell death by targeting multiple members of the antiapoptotic BCL2 family proteins (Fig. 7).

## Discussion

Genomic alterations in splicing genes and splicing dysregulation of cancer-associated genes have been proposed as new hallmark features in cancer^9,37^. Therefore, the development of splicing modulators for cancer treatment is of great interest, exemplified by the SF3b-targeting small molecule splicing modulators^12,13^ and sequence-specific RNA-targeting nucleic acids^38,39^. Although the clinical stage SF3b-targeting splicing modulator H3B-8800 exhibits a preferential killing of spliceosome-mutant cancer cells potentially due to selective retention of short, GC-rich introns^13^, other small molecules modulating the spliceosome machinery may have a more pleiotropic effect on splicing regulation. Thus it is conceivable that a variety of mechanisms of action could underlay splicing modulator-induced preferential cytotoxicity depending on the mechanism of small molecule splicing modulation, genomic traits, and/or splicing-related status in cancer cells. Recently, spliceosome mutation-relevant splicing abnormality, MYC-driven splicing burden, and MCL1 oncogene addiction have been proposed to confer preferential tumor cell inhibition for SF3b-targeting splicing modulators^9,14,15^. In this report, using an unbiased genetic screen, cell line drug sensitivity profiling, and RNA-seq, we identified a differential sensitivity to splicing modulation among five BH-domain *BCL2* family genes, which proposes mechanism-based single-agent and combination therapeutics for SF3b modulators in cancer treatment. We demonstrate that *BCL2A1* and *MCL1* genes are sensitive, whereas *BCL2L1* (BCLxL) is highly resistant to the SF3b modulator E7107. The differential sensitivity to splicing modulation could be associated with intron length and GC content of these genes^17,40^. Consequently, E7107 induces preferential cytotoxicity in BCL2A1-high/dependent melanoma cells and MCL1-high/dependent NSCLC cells. Furthermore, combination of E7107 with BCLxL inhibitors enhances the cytotoxicity in a variety of cancer cell lines. Therefore, in addition to the previously proposed biomarker selection and patient stratification ideas, we further expand the potential clinical applications of SF3b-targeting splicing modulators.

Evading apoptosis is a hallmark of cancer development^41^, which can be co-opted in malignant cells via dysregulation of a number of molecular pathways. One such route frequently utilized by cancer cells is upregulation of the antiapoptotic BCL2 family proteins, including BCL2, MCL1, BCL2A1, and BCLxL. Therefore, the development of strategies targeting these pro-survival factors have been a central theme for cancer drug development. The recent FDA approval for the BCL2-specific inhibitor Venclexta (ABT-199/GDC-0199) in chronic lymphocytic leukemia (CLL) provided clinical proof of concept for such approaches. However, development of small molecules directly targeting other BCL2 proteins has been challenging despite recent significant progress^42^. Moreover, cancer cells tend to employ more than one antiapoptotic BCL2 family proteins to promote cancer cell survival. Therefore, the application of BCL2 family protein-specific inhibitors often encounters drug resistance mediated by induced overexpression of other BCL2 proteins^43^. Clinical and preclinical observations indicate that higher expression of MCL1 and BCL2A1 would render BCL2/BCLxL inhibitors, e.g., ABT199 and ABT263, ineffective, highlighting the need to combine BCL2/BCLxL inhibitors with MCL1 or BCL2A1 inhibitor. Here we show that splicing modulators, such as E7107, can efficiently modulate both *MCL1* and *BCL2A1* and therefore serve as an ideal combinational partner with BCL2/BCLxL inhibitors. This combination strategy may offer effective repression of most of the cancer-relevant antiapoptotic BCL2 family members, thus broadening the impact of both compounds to a variety of indications and overcome the resistance to the current BCL2/BCLxL-targeting agents.

It has been challenging to identify mechanism-based cancer therapeutic strategies for entities targeting essential cellular pathways. The translational approaches we applied here for E7107, combination of genetic functional screen upon drug treatment, drug sensitivity screen in a large panel of omics-characterized cancer cell lines, and unbiased molecular profiling of the key pathway-targeting node of the drug, in our case the RNA-seq for splicing modulation, may help to shed light on the potential clinical development strategies. Indeed, one of the findings in our report that MCL1 and BCLxL could form a robust pair of “lethal partners” has been independently suggested by other genetic screenings^44–46^. The preference to BCLxL rather than BCL2 inhibitor in combination with E7107 may also reflect the selective insensitivity of *BCL2L1* (BCLxL) to splicing modulation (Fig. 3). These findings suggest that an enhanced cytotoxicity can be achieved by targeting both MCL1 and BCLxL in some cancer types. The development of BCLxL-selective inhibitors has been hampered owing to on-target toxicity in patients, e.g., thrombocytopenia and T cell lymphopenia^47,48^. Therefore, the therapeutic window of splicing modulator/BCLxL inhibitor combination needs to be carefully investigated in future preclinical or clinical studies. In any case, our approaches and findings reveal potential cancer therapeutic strategies, single agent or combination, for some SF3b-targeting splicing modulators based on a molecular mechanism underlying differential sensitivity of *BCL2* family genes to splicing modulation.

## Methods

### Cell lines

Cell lines were obtained from the American Type Culture Collection (ATCC) and cultured as per the manufacturer’s instruction. The mutation status of spliceosome genes *SF3B1*, *SRSF2*, and *U2AF1* of all cell lines used in the study is shown in Supplementary Data 5. Inducible shRNA and cDNA lines generated by lentiviral transduction were cultured according to the manufacturer’s instructions utilizing Tet System Approved fetal bovine serum (Clontech) rather than standard sera. The isogenic pair cell lines (Nalm-6 SF3B1^K700E^, and Nalm-6 SF3B1^K700K^, referred as SF3B1^WT^) were published previously^49^. All cell lines have been tested for mycoplasma contamination and authenticated to confirm cell identity by whole-exome sequencing. LentiX-293T (Clontech) was used for the generation of shRNA virus for infection and cultured according to the manufacturer’s instruction.

### Cell line engineering with cDNA or shRNA

BCLxL shRNA 1 (GCTCACTCTTCAGTCGGAAAT)^33^ was cloned into AgeI and EcoRI of the Tet-inducible lentiviral pLKO-iKD-H1 puro vector^50^. MCL1 shRNA 48 (GCATCGAACCATTAGCAGAAA)^33^ was cloned into AgeI and EcoRI of the Tet-inducible lentiviral pLKO-iKD-U6 puro vector, modified from pLKO-iKD-H1 puro vector where the H1 promoter was excised and U6 cloned in. MCL1-L cDNA (EX-Y4182-Lv105, Genecopoeia) was PCR amplified using MCl1-pENTR D TOPO.KOZAK.v1-F (CACCATGTTTGGCCTCAAAAGAAAC) and MCl1-L D TOPO.v1-R (CTATCTTATTAGATATGCCAAACCAGC) primers using PfuUltra II HotStart Master Mix (Agilent) and cloned into pENTR-D-TOPO. MCL1-L pENTR-D-TOPO and BCL2A1 variant 1 pDONR (GeneCopoeia GC-I0365) were Gateway cloned (LR clonase, Thermo Fisher Scientific) into pINDUCER20^51^. Lentiviruses were prepared in LentiX-293T cells. Cells were transfected with 2.4 µg of target pLKO-shRNA or pINDUCER20 plasmid, plus 2.4 µg of pΔ8.91 (packaging), and 0.6 µg VSVG (envelope) using TransIT reagent (Mirus). pINDUCER20+MCL1-L, pINDUCER20 vector, pLKO-iKD-U6 puro+MCL1 shRNA 48, and pLKO-iKD-U6 puro vector viruses were used to infect A549, NCI-H23, NCI-H1568, NCI-H1650, NCI-H1975, and NCI-H2110. pLKO-iKD-H1 puro+BCLxL shRNA 1 and pLKO-iKD-H1 puro viruses were used to infect A549, NCI-H23, NCI-H1568, and NCI-H2110. pINDUCER20+BCL2A1 and pINDUCER20 vector viruses were used to infect HT144 and COLO829. Cells were infected with or without spin infection using Polybrene (Millipore). One to three days after infection, the cells were cultured in Geneticin (pINDUCER20) (0.5–2 mg ml^−1^, Thermo Fisher Scientific) or Puromycin (pLKO shRNAs) (0.25–1.25 µg ml^−1^, Thermo Fisher Scientific). The selected cells were cultured in the presence or absence of Dox (Sigma) (300 ng ml^−1^) for induction of the shRNA and cDNA. Cells were harvested for protein and RNA 3–5 days post induction. RNA was isolated as in the Reverse transcriptase-qPCR section. Protein extracts were prepared as in the Western blot analysis section.

### Pooled shRNA screen

A pooled shRNA library containing 6500 individually barcoded hairpins targeting 841 different genes (~8 shRNAs per gene) covering a broad range of cellular processes (Supplementary Data 1) were generated by Cellecta and delivered as plasmid pool. Pooled shRNA viruses were generated from the plasmid pool using Lenti-X293T cells (Clontech) and TransIT-LT1 reagent (Mirus) according to the manufacturers’ protocol. For shRNA screen, 24 million NALM6 cells were seeded in replicates and infected with pooled shRNA viruses to achieve an infection rate of 30%. Forty-eight hours post infection, NALM6 cells were selected in 1 µg ml^−1^ puromycin for 4 days to eliminate uninfected cells. After puromycin selection, each replicate was split into two flasks with 10 million cells each and treated with either DMSO or 5 nM E7107 for 3 days. At the end of the 3-day treatment, 1 million cell pellets were collected from each sample group and subjected to genomic DNA extraction using the DNeasy Blood and Tissue Kit (Qiagen) as per the manufacturer’s protocol. Integrated hairpin barcodes were amplified from genomic DNAs using PfuUltra II Fusion HS DNA Polymerase (Agilent) in parallel PCR reaction (12, 50 µl reaction per sample) to maintain the representation of the shRNA library. To allow multiplex sequencing on Miseq, unique sample index barcode was built into each reverse PCR primer. Illumina sequencing-compatible PCR product from parallel reactions were combined and purified using the QIAquick PCR Purification Kit (Qiagen). Purified PCR product from each sample was measured using the Qubit™ dsDNA HS Assay Kit (Thermo Fisher Scientific) and combined for the final pool. Following NaOH denaturation, 4 pM of the final pool was loaded onto Miseq for NGS using the Miseq V3 Reagent Kit (Illumina). Bowtie 1 (http://bowtie-bio.sourceforge.net/index.shtml) is used to align the sequenced barcodes to the known library barcode sequences to represent the corresponding shRNAs. The representation of shRNA i is quantified by counting the number of shRNA i presented in the library as ni. Count per ten million (CPTM) of shRNA i is calculated by (ni/*N*_total_)10^6^, where *N*_total_ is the total number of shRNA counts in the respective samples. Log count of a shRNA is calculated by logCPTM + 1, where 1 is the pseudo count. Log ratio of each shRNAs is then generated based on the log ratio values. Hit calling is done by applying moderated *t* test from R limma package.

### Cell line panel drug sensitivity screen

E7107 was tested in a cell line panel containing 478 cancer cell lines with RNA-seq data available from Cancer Cell Line Encyclopedia (Supplementary Data 3). Staurosporine and bortezomib were included to serve as the internal cytotoxic agent controls. CellTiter-Glo luminescent assay was used to measure cell viability in 384-well plates. Each compound was tested for 11 concentration points in triplicate at starting concentrations 10 µM and three-fold serial dilution. Assay-ready plates with 50 nl of the compounds were pre-dispensed in each well of the 384-well plates. The incubation time for the cell lines was 96 h or at least two doubling times for slow-growing cell lines. The day 0 signal (T0) for each cell line was read in a separate plate before compound addition. Quality of the run will be determined by the fold growth of the cell lines and by the response to Staurosporine (GI50 and LD50), and all *Z*’ Factor of the assays were >0.6. The drug sensitivity screen was conducted by Chempartner (Shanghai, China) using the contract research organization services. All the raw data was uploaded into and analyzed in Ecabia (H3 Biomedicine). GI_50_ (the concentration that causes 50% growth inhibition), GI_90_ (the concentration that causes 90% growth inhibition), LD_50_ (the concentration that causes −50% cell death compared to T0), and Emax (the maximal effect, cell killing or growth inhibition, compared to T0) were calculated using Ecabia. The Emax values were represented by minus percentage (−100% to 0%), in which the lowest number (−100%) represents the highest killing, whereas the highest number (0%) represents the lowest killing. The Pearson’s correlation coefficient *R* and *p* values between gene expression and Emax were calculated using R package Hmisc 4.1-0 in R v3.2.3 statistical environment.

### RNA-seq and splicing analysis

The RNA-seq data were generated by the Illumina HiSeq RNA Sequencing platform^17^. The transcript abundance was estimated using Kallisto v0.42.4. Gencode v19 long non-coding RNA transcript sequences downloaded from GENCODE (www.gencodegenes.org) plus custom transcripts of *BCL2, BCL2A1*, *BCL2L1*, *BCL2L2*, and *MCL1* (Supplemental Table 4) were used to build the index. The transcripts abundance was summarized to gene-level isoform abundance. The log2-transformed transcripts per million data were reported. Estimated counts of transcripts from Kallisto were summarized to gene-level isoform abundance and transformed to log2 counts per million (logCPM) using voom in R package limma 3.34.8. The adjusted *p* values were then calculated by using limma. All of the statistical analysis of RNA-seq were done in R v3.2.3 statistical environment (www.r-project.org).

### Reverse transcriptase qPCR

For shRNA experiments, RNA was purified from cell lines using RNeasy Mini with DNaseI treatment (Qiagen) and 1–2 µg of RNA reverse transcribed using Superscript VILO reverse transcriptase (Thermo Fisher Scientific) in 20 µl according to the manufacturer’s instruction. For cells treated with compounds in 96-well plates, RNA lysates were isolated and reverse transcribed using the TaqMan Gene Expression Cells-to-CT Kit (Thermo Fisher Scientific) according to the manufacturer’s instructions. Quantitative PCR was performed using TaqMan Gene Expression Master Mix (Thermo Fisher Scientific) with MCL1 transcript probes (Integrated DNA technologies FAM-ZEN/IBFQ) duplexed with 18S rRNA VIC-PL (Thermo Fisher Scientific assay ID Hs99999901_s1) and quantified using the *ΔΔCt* method. Taqman gene expression probes used in these assays are listed below (Table 1).

**Table 1:** Taqman gene expression primer and probe sequences

MCL1-L probe set	
Forward primer	ATATGCCAAACCAGCTCCTAC
Probe	AGAACTCCACAAACCCATCCCAGC
Reverse primer	AAGGACAAAACGGGACTGG
MCL1-S probe set	
Forward primer	AAAGCCAATGGGCAGGT
Probe	TCCACAAACCCATCTTGGAAGGCC
Reverse primer	CCACCTTCTAGGTCCTCTACAT
MCL1 intron1 probe set	
Forward primer	GACAAAGGAGGCCGTGAGGA
Probe	TCAGGCATGCTTCGGAAACTGGA
Reverse primer	GTTTGTTACGCCGTCGCTGAAA
MCL1 intron2 probe set	
Forward primer	GCCCCGGGGTGAATAATAATTGGTTTACT
Probe	TTTCTAGGATGGGTTTGTGGAGTT
Reverse primer	CCTGATGCCACCTTCTAGGTCCTCTAC
Pan MCL1 probe set	
Forward primer	GCCAAGGACACAAAGCCAAT
Probe	CTGGAGACCTTACGACGGGTTGGG
Reverse primer	AAGGCCGTCTCGTGGTT
Pan BCL2A1 probe set	
Forward primer	AGTCATGCTTGGACAATGTTAATG
Probe	TGTCCGTAGACACTGCCAGAACAC
Reverse primer	GATGCCGTCTTCAAACTCCT
BCL2A1 exonic probe set	
Forward primer	GGATGTGGATACCTATAAGGAGATTT
Probe	AAACGGAGGCTGGGAAAATGGCTT
Reverse primer	AAGTCATCCAGCCAGATTTAGG
BCL2A1 intronic probe set	
Forward primer	GCTGGGTATGTGTGATGGAA
Probe	TTTCCTTGGGTGGTTTGTTT
Reverse primer	GGATTCATTGGAAATAAGCCAGAA

### Western blot analysis

Cell lines were lysed in RIPA buffer (Boston BioProducts) plus protease-inhibitor cocktail (Mini-complete, EDTA-free, Roche) and phosphatase inhibitor PhosSTOP (Roche). Lysates were diluted in RIPA buffer with 4× LDS Sample Buffer (Nupage, Thermo Fisher Scientific) and 10× Reducing Reagent (Nupage, Thermo Fisher Scientific) and boiled for 5 min. Twenty-five micrograms of protein were loaded per well in 4–12% Bis-Tris sodium dodecyl sulfate Page gels (Novex, Thermo Fisher Scientific). Gels were transferred to nitrocellulose membranes using iBlot system (Thermo Fisher Scientific). Membranes were blocked in blocking buffer (1× Tris-Buffered Saline + 0.1% Tween-20 (Boston Bioproducts) + 5% Non-Fat Dry Milk (Bio-Rad)) for 1 h and then cut. Each section was probed separately with antibodies to each of the following proteins in blocking buffer: MCL1 (Cell Signaling Technologies 5453) (D35A5) rabbit monoclonal diluted 1:500, BCLxL (Cell Signaling Technologies 2764) (54H6) rabbit monoclonal diluted 1:500, BCL2 (Cell Signaling Technologies D55G8) (4223) rabbit monoclonal diluted 1:500, BCL2L2/BCLw (R&D Systems AF824) rabbit polyclonal diluted 1:500, Cleaved Parp1 (Cell Signaling Technologies 5625) (D64P10) rabbit monoclonal diluted 1:500, Tubulin (Sigma T6199) mouse monoclonal at 50 ng ml^−1^, and GAPDH (Sigma G8795) mouse monoclonal at 100 ng ml^−1^. Blots were incubated with primary antibodies dilutions shaking at 4 °C overnight. Western blots were then blotted either with Odyssey Licor or with horseradish peroxidase (HRP) secondary antibodies. Blots were then washed four times using wash buffer (1× Tris-Buffered Saline + 0.1% Tween-20). Blots imaged using Licor were probed shaking at room temperature for 1 h with Licor IR-labeled secondary antibodies, IRDye® 800CW Goat anti-Rabbit IgG (Odyssey 925-32211) and IRDye® 680LT Goat anti-Mouse IgG (Odyssey 925-68020) diluted 1:10,000 in blocking buffer. Blots were then washed three times with wash buffer. IR-dye detection was performed using the Licor imaging system (Odyssey) according to the manufacturer’s instruction. Blots imaged using HRP were probed shaking at room temperature for 1 h with Anti-mouse IgG, HRP-linked Antibody (Cell Signaling Technologies 7076), and Anti-rabbit IgG, HRP-linked Antibody (Cell Signaling Technologies 7074) diluted 1:5000 in blocking buffer. Blots were then washed three times with wash buffer. HRP detection was performed using SuperSignal West Femto Maximum Sensitivity Substrate (Thermo Fisher Scientific) and imaged with ImageQuant™ LAS 4000 biomolecular imager (GE Healthcare Life Sciences) according to the manufacturer’s instruction. Uncropped images of western blots and gels are shown in Supplementary Fig. 13.

### GFP-tracking viability assay

To validate the results from pooled shRNA screen, 5 hairpins against *BCL2L1* that showed sensitizing effect to E7107 treatment corresponding to barcode IDs 251, 252, 253, 255, and 5084 (Supplementary Data 1) as well as a negative control shRNA against luciferase were individually cloned into a lentiviral vector containing GFP tag by Cellecta. shRNA viruses were generated from each plasmid using Lenti-X293T cells (Clontech) according to the manufacturer’s instruction. For GFP-tracking viability assay, NALM6 cells were seeded into 96-well plate and infected with individual shRNA virus at ~50% infection rate. After overnight infection, media was refreshed with normal complete media. Three days post infection, NALM6 cells were split into three separate plates with one plate used as Day 0 controls by measuring the GFP-positive percentage via flow cytometry, whereas other two plates were treated with either DMSO or 5 nM E7107. Three days post compound treatment, both plates were assayed for GFP-positive percentage by flow cytometry.

### Cell viability assay

For inducible shRNA experiments, cells were cultured in 96-well with or without 300 ng ml^−1^ Dox for 72 h and then lysed with CellTiter-Glo Luminescent Cell Viability Assay (Promega) according to the manufacturer’s instruction and analyzed using Perkin-Elmer Envision. Dox-treated wells were normalized to no treatment for each cell line. For compound dose–response experiments, cells were plated in 96- or 384-well plates. Compounds were serial diluted and added to cells in media with 0.1% final DMSO. At *t* = 0, CellTiter-Glo was read for untreated cells and 72 h post compound addition (*t* = 72) and analyzed on Envision. For MCL1 and BCL2A1 rescue experiments, cell lines were cultured for 72 h with 300 ng ml^−1^ Dox, then sub-cultured and seeded into 96- or 384-well plates with 300 ng ml^−1^ Dox.

### Caspase activation and apoptosis assay

Cells infected with pINDUCER20 vector or cDNA overexpression (MCL1L, BCL2A1) were induced for 3 days in 300 ng ml^−1^ Dox, then seeded in 96-well clear bottom plates (Corning, #3904) with IncuCyte Caspase-3/7 Green Apoptosis Assay Reagent (Cat No 4440). The following day, cells were treated with E7107 and monitored by HD phase-contrast imaging and green fluorescence imaging every 2 h with the IncuCyte ZOOM System (Essen BioScience) using 10× objective lens. Collected images were analyzed with the IncuCyte ZOOM Software (2015A) (Essen BioScience) to calculate the confluence percentage and total green intensity. Caspase activation was calculated 24 h (MCL1L) or 72 h (BCL2A1) by dividing total green intensity by percentage of confluence.

### Drug combination assay

Compound serial dilutions were performed as in the Cell viability assay section. For BCLxL knockdown, cells were cultured with 300 ng ml^−1^ Dox for 3 days as per the Cell viability assay section prior to seeding 384-well plates. CellTiter-Glo was performed as per the Cell viability assay section. Data were analyzed using Chalice (Horizon Discovery). To estimate compound synergy, we used Horizon Discovery’s Loewe Additivity model, where Loewe Excess is calculated by subtracting the Loewe Model (pure additivity based on compound self-cross) from the Dose–Response matrix.

### In vivo xenograft tumor study

NCr nude female mice were sourced from Charles River (Wilmington, MA) and maintained in a pathogen-free facility according to Institutional Animal Care and Use Committee (IACUC) guidelines. All animal experiments were conducted according to IACUC guidelines defined by the H3 Biomedicine Animal Care and Use Program and study protocol. Animals were implanted with tumor cells at 6–8 weeks of age. For each model, 10 × 10^6^ cells were implanted in 50% Matrigel (Corning) subcutaneously in the flank. Tumors were randomized at 150–200 mm^3^ and dosed for 5 consecutive days as indicated in the text. E7107 was formulated in 10% Ethanol, followed by 5% Tween20 and qs with saline. Tumor measurements using calipers were taken every 3 days, and tumor volume was calculated according to the following formula: *V* = (*L* × *W*^2^)/2. For Dox studies (Sigma), animals were implanted as indicated above and placed on Dox chow (1000 mg kg^−1^, Lab Nutrients) for 3 days before dosing. Statistical significance was analyzed by the Holm–Sidak method in Prism (GraphPad Software, La Jolla, CA).

### Statistical analysis

Appropriate statistical methods were performed as described in specific Methods sections. *p* < 0.05 was considered as statistically significant.

### Reporting Summary

Further information on experimental design is available in the Nature Research Reporting Summary linked to this article.

## Supplementary information

Supplementary Information

Description of Additional Supplementary Files

Supplementary Data 1

Supplementary Data 2

Supplementary Data 3

Supplementary Data 4

Supplementary Data 5

Reporting Summary

## Data Availability

RNA-seq data have been deposited in NCBI’s BioProject database and are accessible through BioProject ID PRJNA509041. The authors declare that all the other data supporting the findings of this study are available within the article and its supplementary information files and from the corresponding author upon reasonable request.
