# Supplementary material for: Sensitivity to splicing modulation of BCL2 family genes defines cancer therapeutic strategies for splicing modulators
Source: Nat Commun. 2019 Jan 11;10:137. doi: 10.1038/s41467-018-08150-5 (PMC6329755; doi:10.1038/s41467-018-08150-5)
Supplement: Supplementary file 1 — Supplementary Information [file 41467_2018_8150_MOESM1_ESM.pdf]

Sensitivity to Splicing Modulation of *BCL2* Family Genes Defines Cancer Therapeutic Strategies for Splicing Modulators

Aird, Teng, *et al.*

a

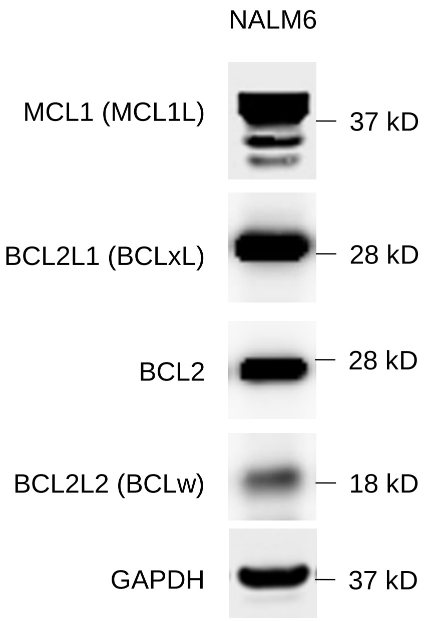

b

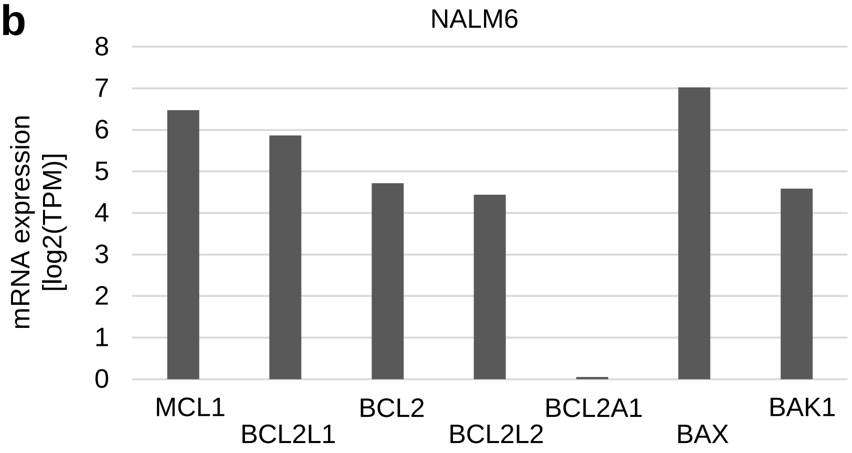

Supplementary Figure 1. Expression of the BCL2 family genes in NALM6 cell line. (a) Western blot analysis of expression of the anti-apoptotic BCL2 family proteins in NALM6 cells. **(b)** RNAseq analysis of expression of the *BCL2* family genes in NALM6 cells.

a

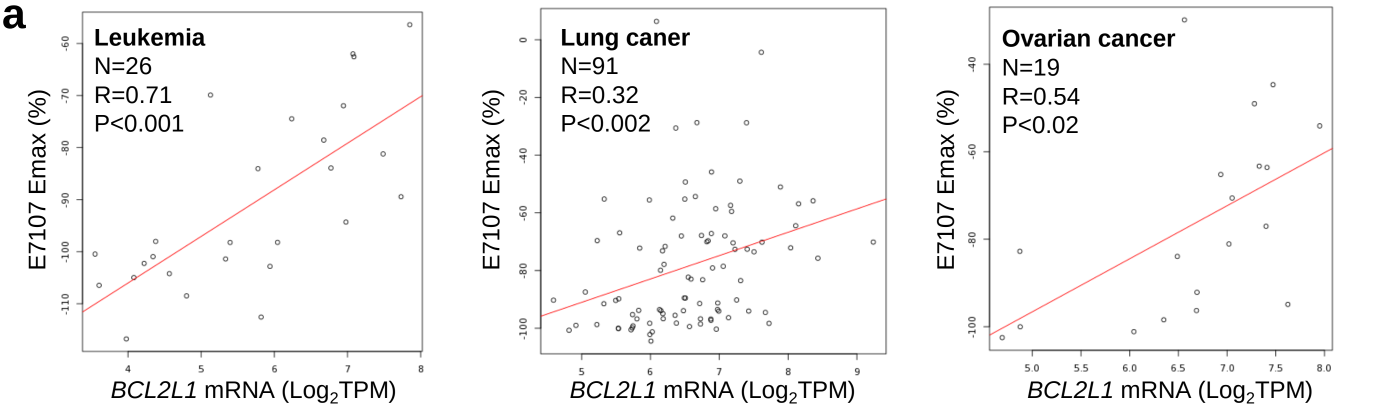

b

gene	r	p	adj.p
BCL2L1	0.385	0	0
CARD10	0.338	0	0
NCEH1	0.336	0	0
ERRFI1	0.334	0	0
ARHGAP29	0.329	0	0

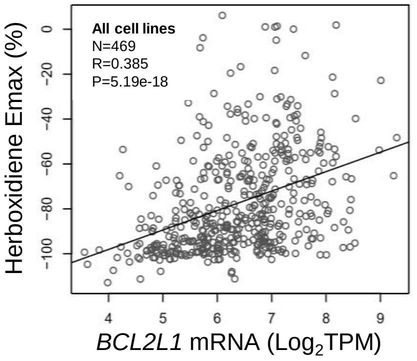

c

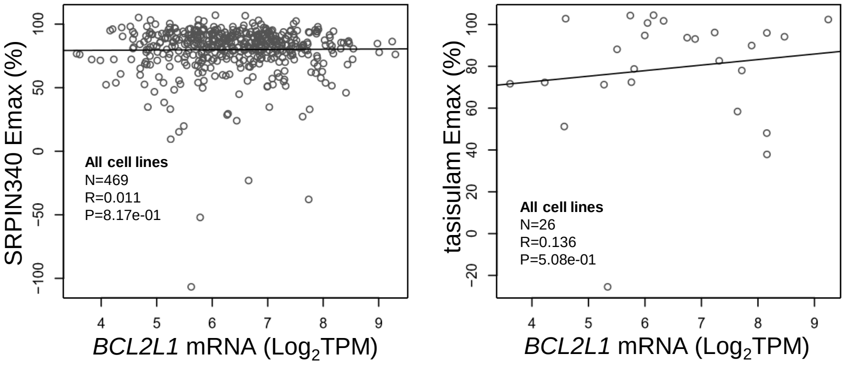

d

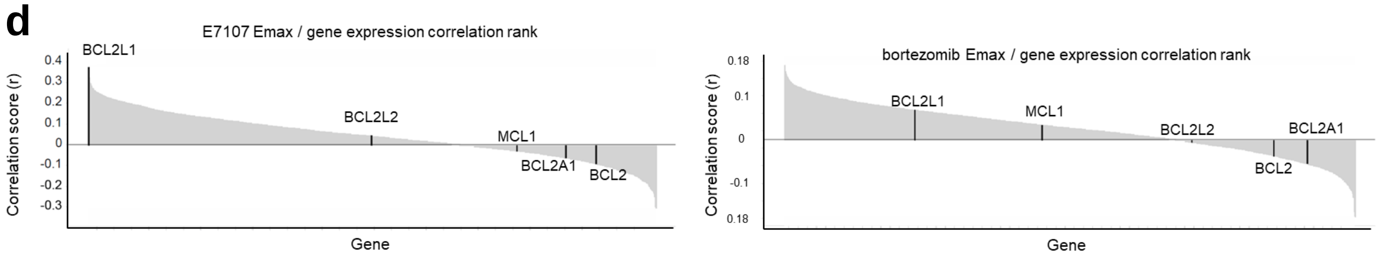

Supplementary Figure 2. Drug sensitivity screen in a 478 human cancer cell line panel reveals *BCL2L1* as a top insensitivity marker for SF3b-targeting splicing modulators, but not other splicing modulators or proteasome inhibitor bortezomib. **(a)** Correlation of E7107 maximum effect of cell killing (Emax) with *BCL2L1* mRNA expression in leukemia, lung cancer and ovarian cancer cell lines. **(b)** *Top*, Top five genes whose mRNA expression positively correlated with the Emax of a structurally different SF3b-targeting splicing modulator herboxidiene profiled in cancer cell lines. *Bottom*, Correlation of the herboxidiene Emax with *BCL2L1* mRNA expression in the cell line panel. **(c)** Correlation of the Emax of SRPK-inhibiting splicing modulator SRPIN340 (left) and RBM39/DCAF15-targeting splicing modulator tasisulam (right) with *BCL2L1* mRNA expression in the cell line panel. **(d)** Waterfall plots showing the rank order of the Pearson's correlation coefficient R in 478 profiled cell lines. *BCL2L1* ranked as the No.1 gene whose expression is positively correlated with E7107 Emax, whereas there is no correlation between bortezomib Emax and *BCL2L1* mRNA expression. Pearson's correlation coefficient R and p-values were calculated using R package Hmisc 4.1-0.

a

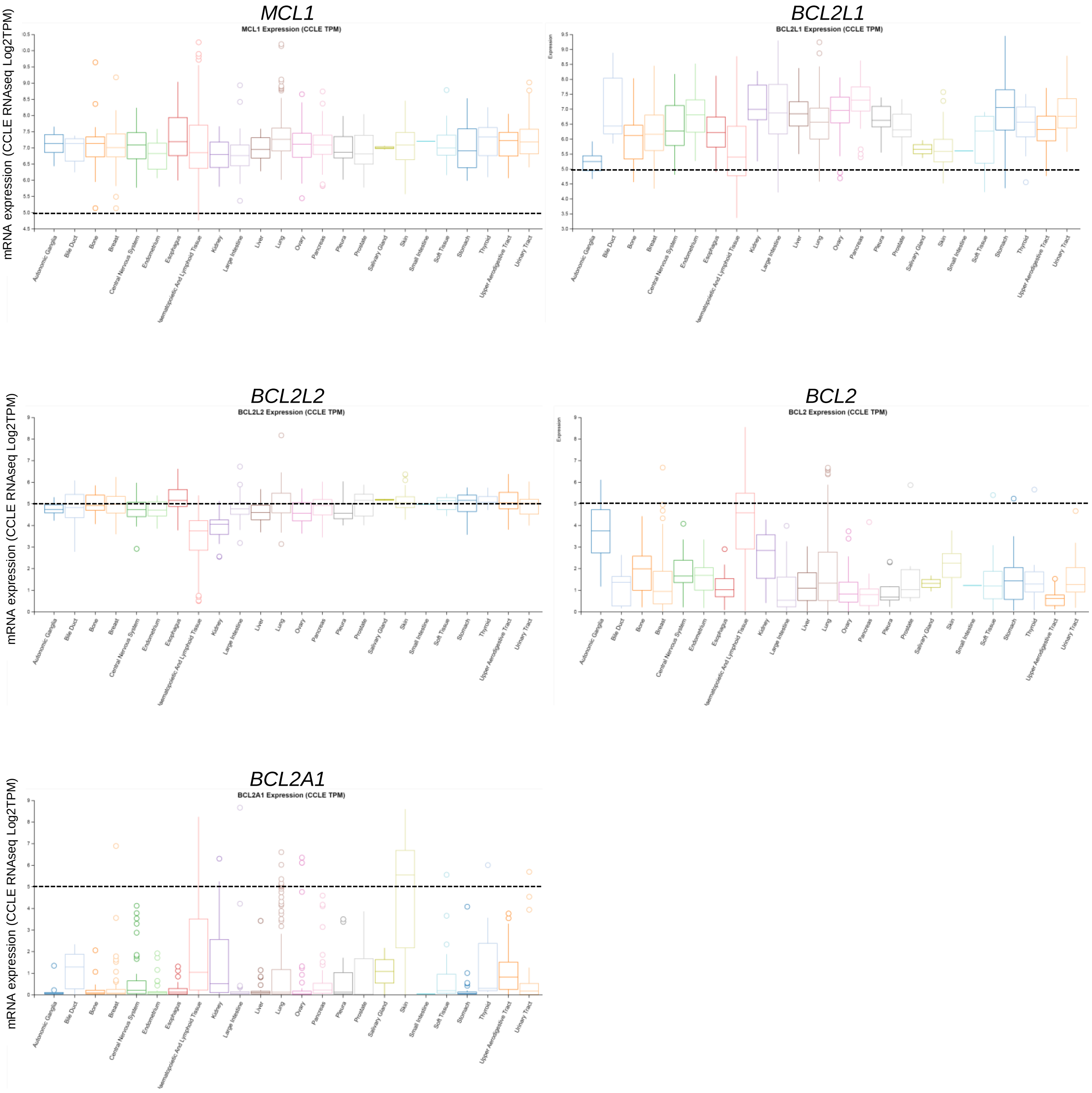
MCL1
MCL1 Expression (CCLE TPM)
BCL2L1
BCL2L1 Expression (CCLE TPM)
BCL2L2
BCL2L2 Expression (CCLE TPM)
BCL2
BCL2 Expression (CCLE TPM)
BCL2A1
BCL2A1 Expression (CCLE TPM)
mRNA expression (CCLE RNAseq Log2TPM)
Expression
Autonomic Ganglia
Bile Duct
Bone
Breast
Central Nervous System
Endometrium
Esophagus
Haematopoietic And Lymphoid Tissue
Kidney
Large Intestine
Liver
Lung
Ovary
Pancreas
Pleura
Prostate
Salivary Gland
Skin
Small Intestine
Soft Tissue
Stomach
Thyroid
Upper Aerodigestive Tract
Urinary Tract

b

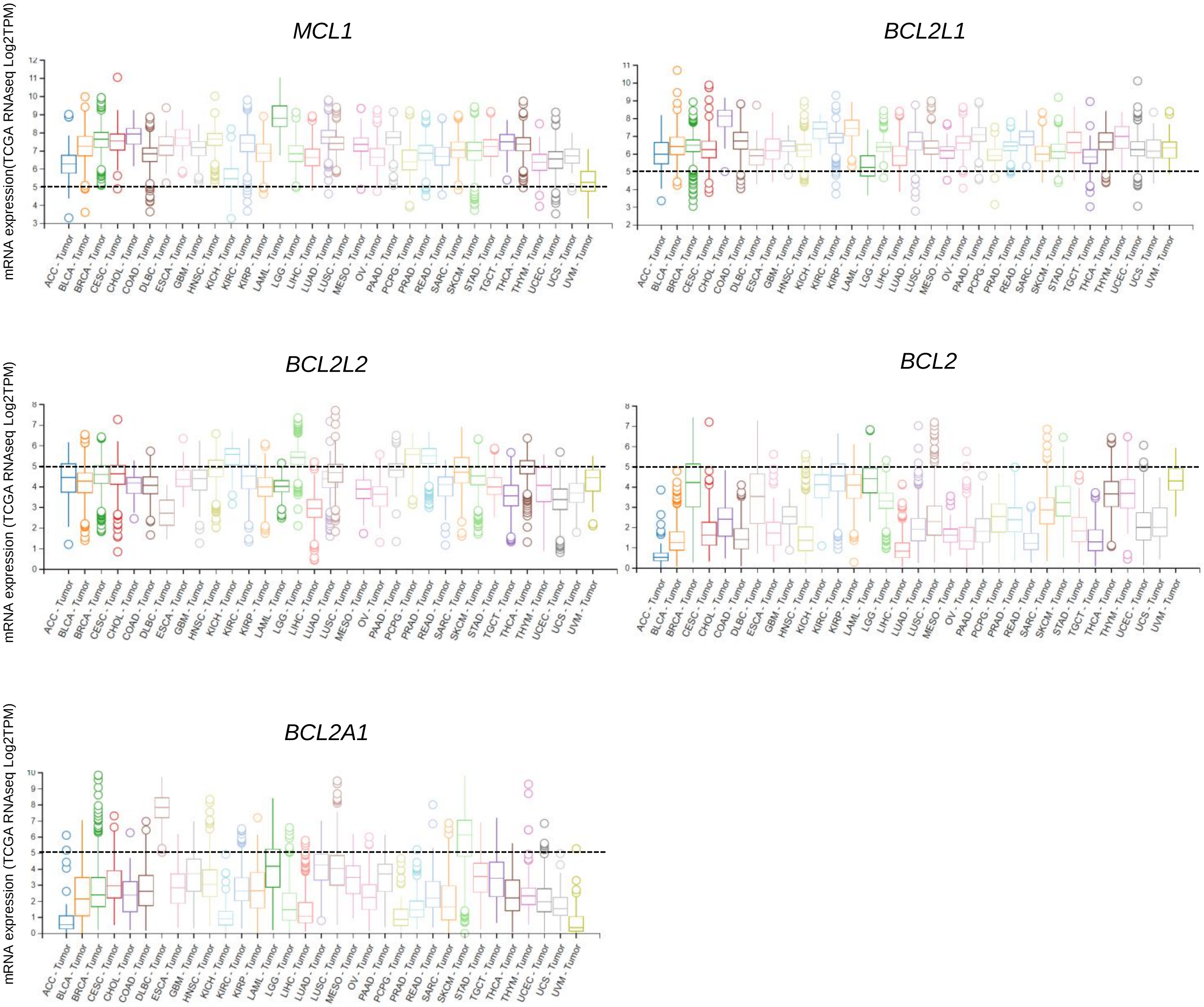

Supplementary Figure 3. mRNA expression levels of five anti-apoptotic BCL2 family genes. (a) Box plots showing mRNA expression distribution in cancer cell lines from a various of lineages/tissue types based on the RNAseq data from Cancer Cell Line Encyclopedia (CCLE). **(b)** Box plots showing mRNA expression distribution in the tumor tissues profiled by The Cancer Genome Atlas (TCGA). Dotted lines represent Log2(TPM) = 5.

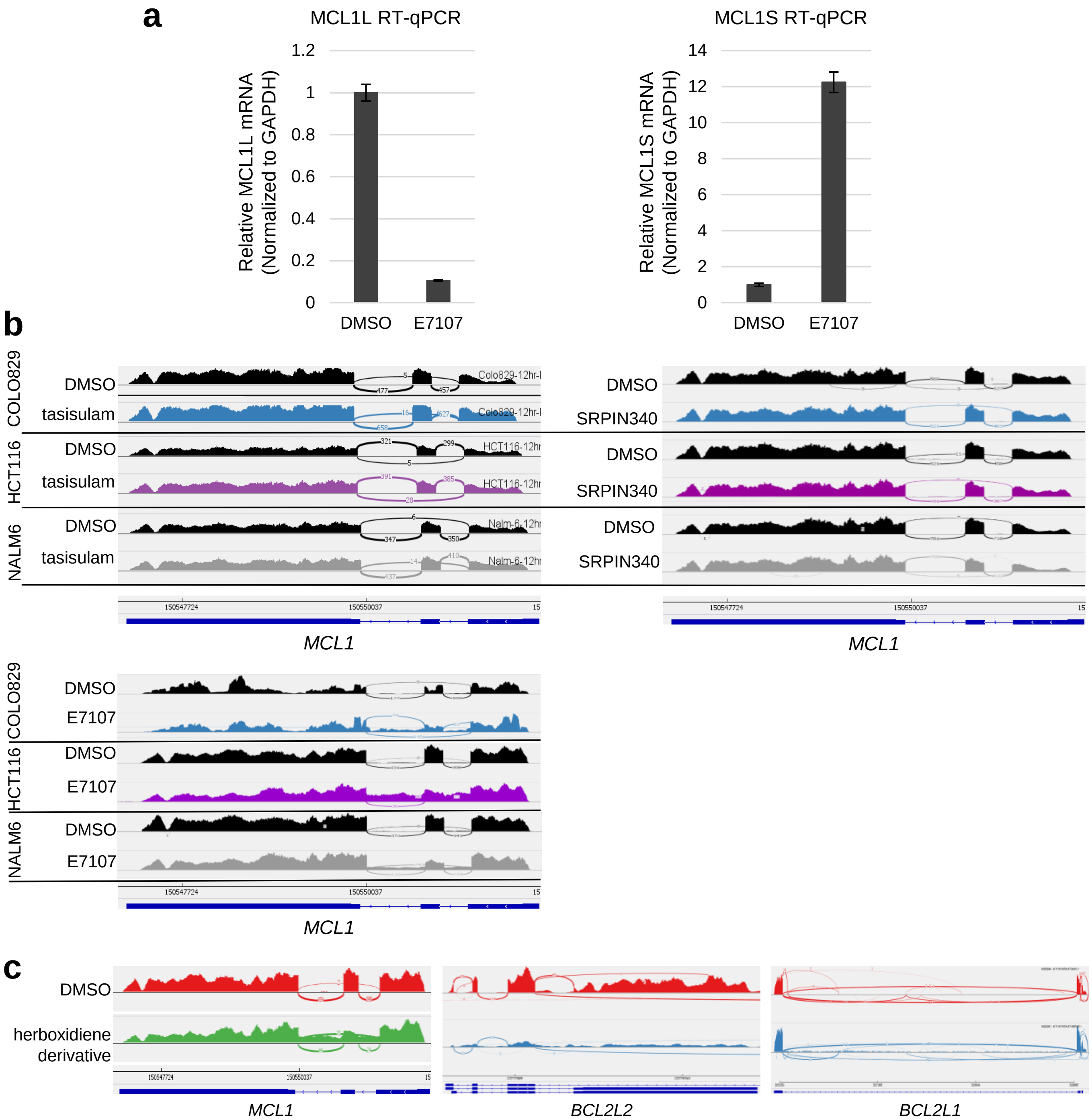

Supplementary Figure 4. Differential splicing modulation of *BCL2* genes by different small molecule splicing modulators. (a) mRNA expression of MCL1L and MCL1S was confirmed by probe-specific RT-qPCR. HCT116 cells were treated with E7107 (50nM for 6hrs) and the mRNA of MCL1L and MCL1S was examined by primers and TaqMan probes specific for MCL1L or MCL1S. **(b)** COLO829, HCT116 or NALM6 cells were treated with RBM39/DCAF15 inhibitor tasisulam (30μM for 12hrs), SRPK inhibitor SRPIN340 (30μM for 12hrs), or SF3b modulator E7107 (50nM for 6hrs), and mRNA samples were collected for RNAseq. While E7107 substantially disrupted *MCL1* splicing, neither tasisulam nor SRPIN340 did. **(c)** HCT116 cells were treated with a SF3b modulator herboxidiene derivative (50nM for 6hrs), and mRNA samples were collected for RNAseq. Similar to E7107, herboxidiene derivative disrupted splicing of both *MCL1* and *BCL2L2*, whereas *BCL2L1* was resistant. Data in (b) and (c) were shown as Sashimi plots with junction reads labeled.

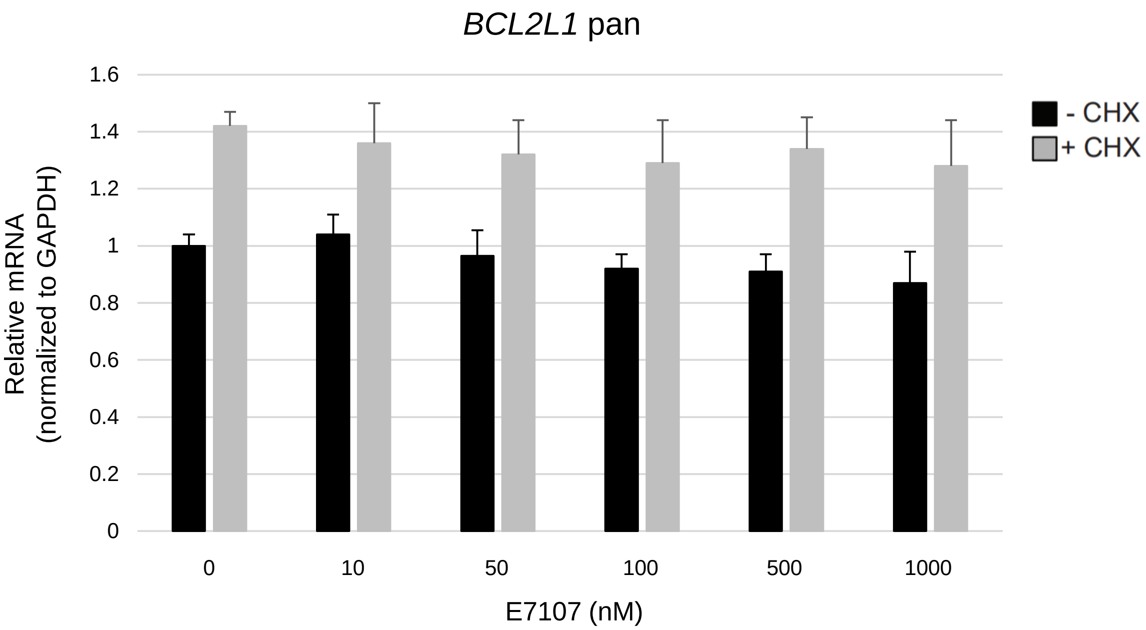

Supplementary Figure 5. mRNA expression of pan *BCL2L1* in HT144 melanoma cell line by RT-qPCR. RT-qPCR analysis of total mRNA of BCL2L1. HT144 cells were pre-treated with 100ug/mL cycloheximide (CHX) for 1 hour, followed by addition of E7107 as indicated for 12 hours to inhibit nonsense-mediated mRNA decay (NMD) before collecting RNA samples.

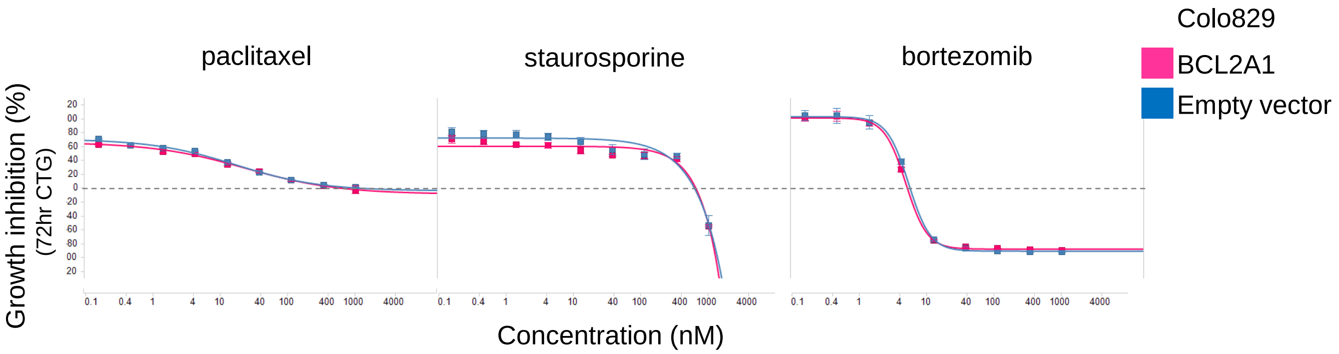

Supplementary Figure 6. BCL2A1 expression does not rescue the cytotoxicity induced by proteasome inhibitor bortezomib and pan-kinase inhibitor staurosporine. Growth curves measured by CellTiter-Glo (CTG) in Colo829 melanoma cell lines stably transduced with vector control (empty vector) or BCL2A1 cDNA (BCL2A1) treated with paclitaxel, staurosporine or bortezomib for 72 hours.

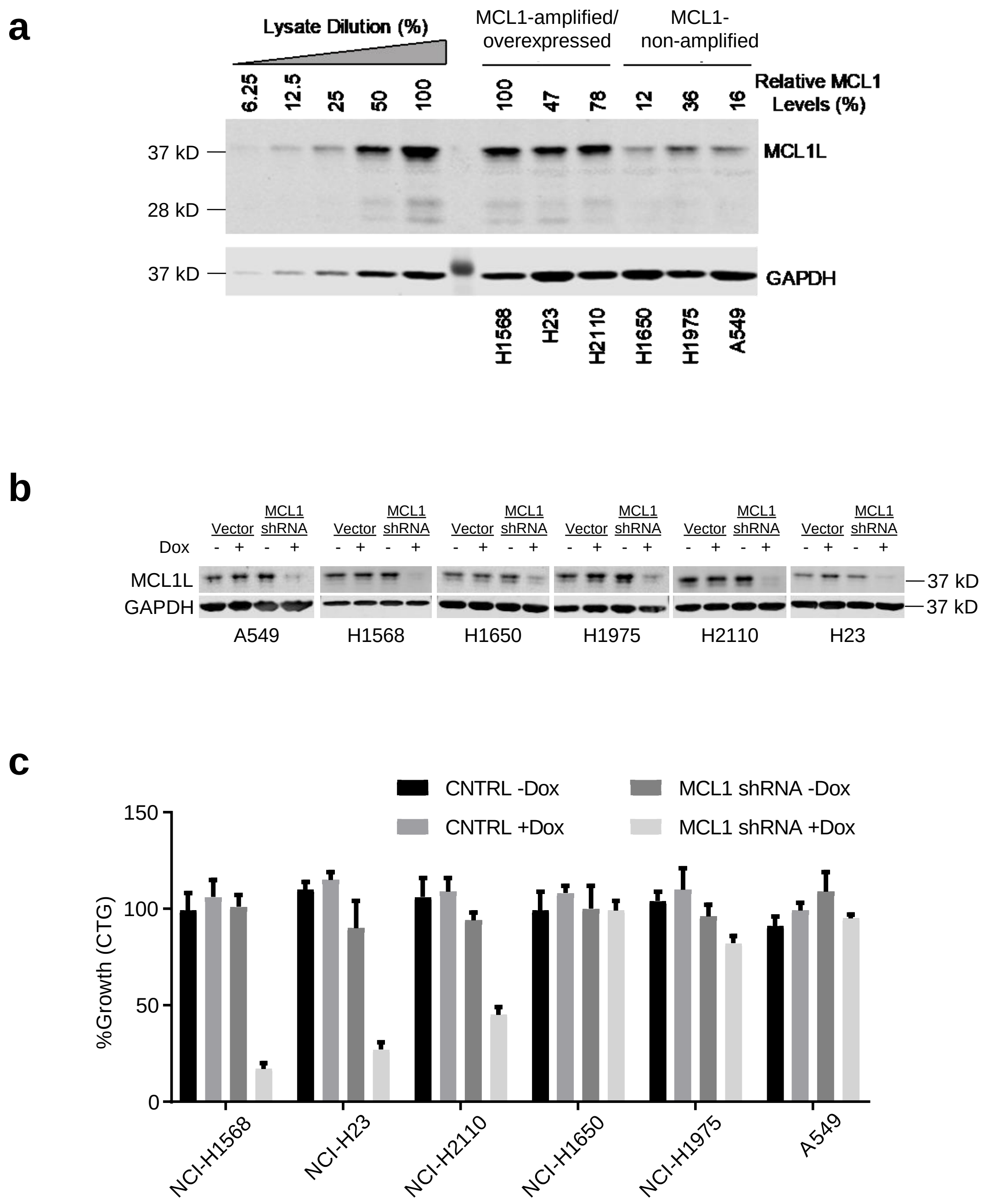

Supplementary Figure 7. Validation of MCL1-high/dependent and MCL1-low/independent NSCLC cell lines. (a) Western blot analysis of MCL1 and GAPDH (loading control). Serial dilution of samples was used a semi-quantification method to calculate the relative MCL1 expression levels normalized to GAPDH. **(b)** Western blot analysis for NSCLC cell lines engineered with doxycycline (Dox) inducible control vector (Vector) or MCL1 shRNA after 24h (Amplified, MCL1 Dependent) or 72h (Non-Amplified, MCL1 Independent) Dox treatment. **(c)** Viability assay (CTG) for NSCLC cell lines engineered with doxycycline (Dox) inducible control vector (CNTRL) or MCL1 shRNA after 96h Dox treatment.

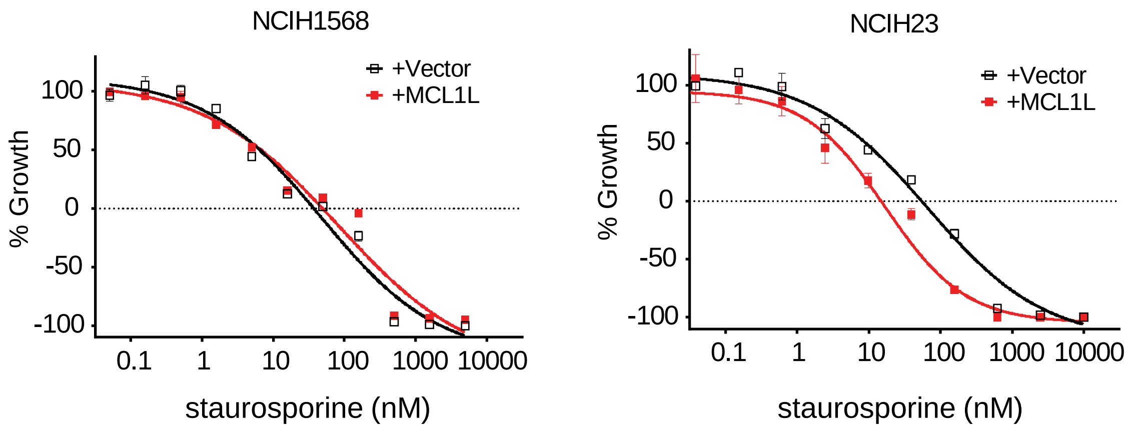

Supplementary Figure 8. MCL1L expression does not rescue the cytotoxicity induced by pan-kinase inhibitor staurosporine. Growth curves measured by CellTiter-Glo (CTG) in MCL1-dependent NSCLC cell lines stably transduced with vector control (Vector) or MCL1L cDNA (MCL1L) treated with staurosporine for 72 hours.

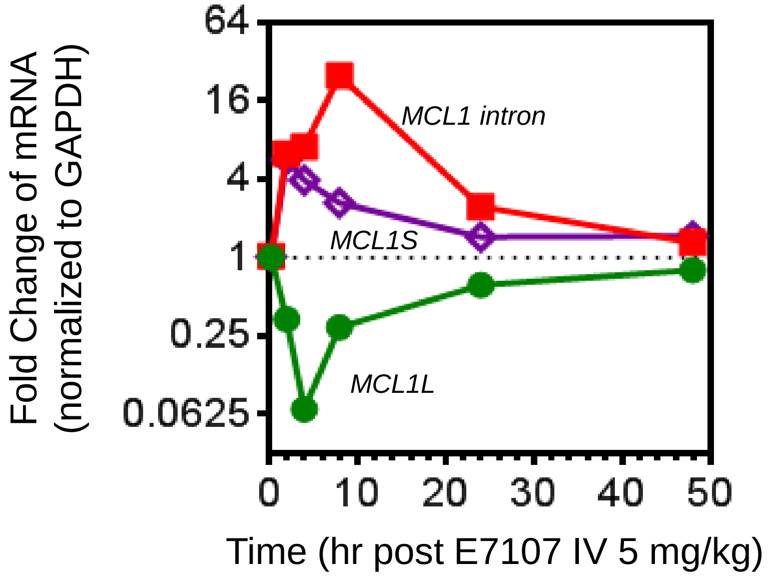

Supplementary Figure 9. E7107 reduces MCL1L and induces MCL1S and intron-retention transcripts in the NCIH1568 xenograft tumor model. RT-qPCR analysis of MCL1 transcripts with different splicing variants in the NCIH1568 xenograft tumors upon single dose (IV 5 mg/kg) treatment with E7107.

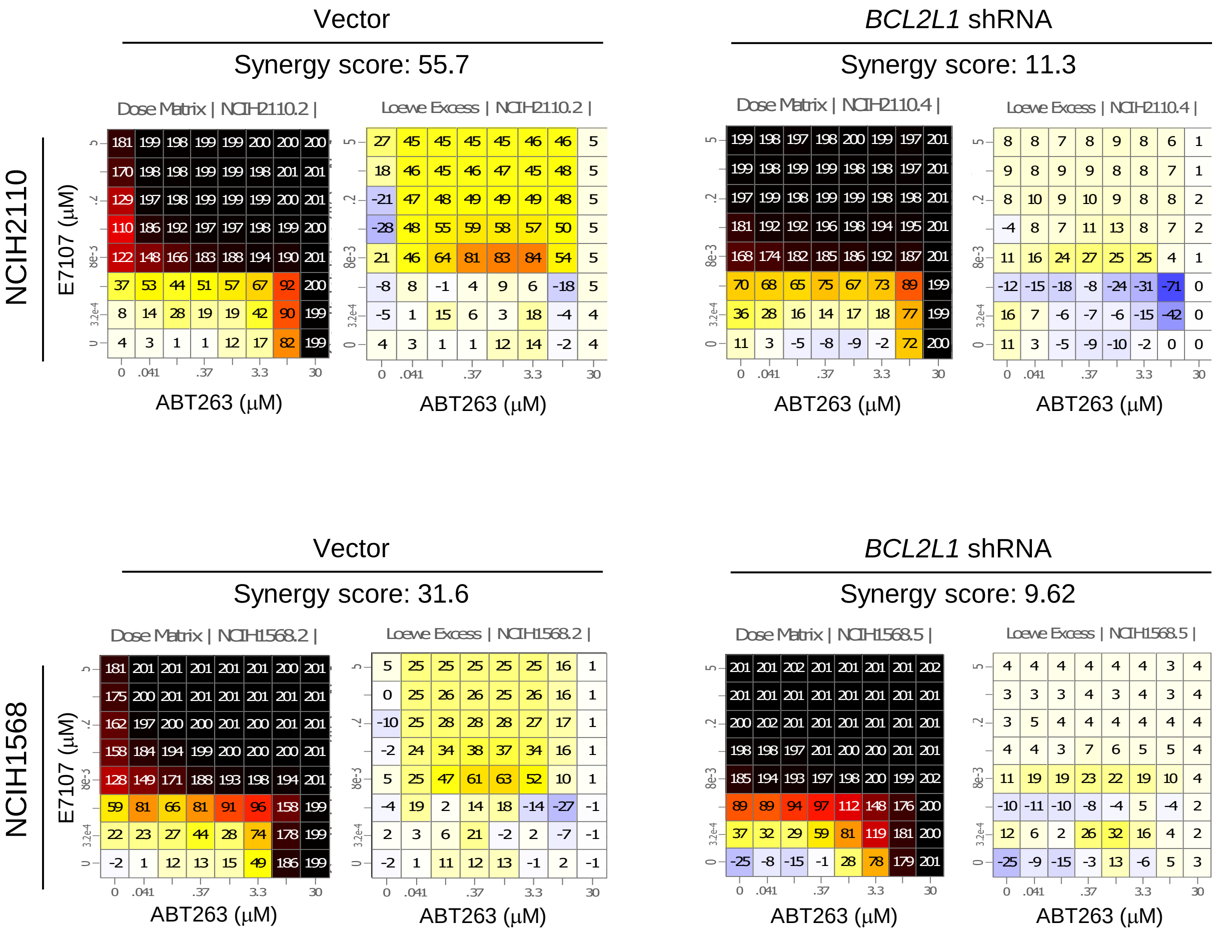

Supplementary Figure 10. Combination of E7107 and ABT263 induces synergistic cytotoxicity in NSCLC cell lines. 8x8 dose matrix combination study for E7107 and ABT263 in MCL1-dependent NCIH2110 and NCI1568 cell lines stably transduced with vector control (Vector) or *BCL2L1* shRNA. Synergy scores were calculated by the Chalice software.

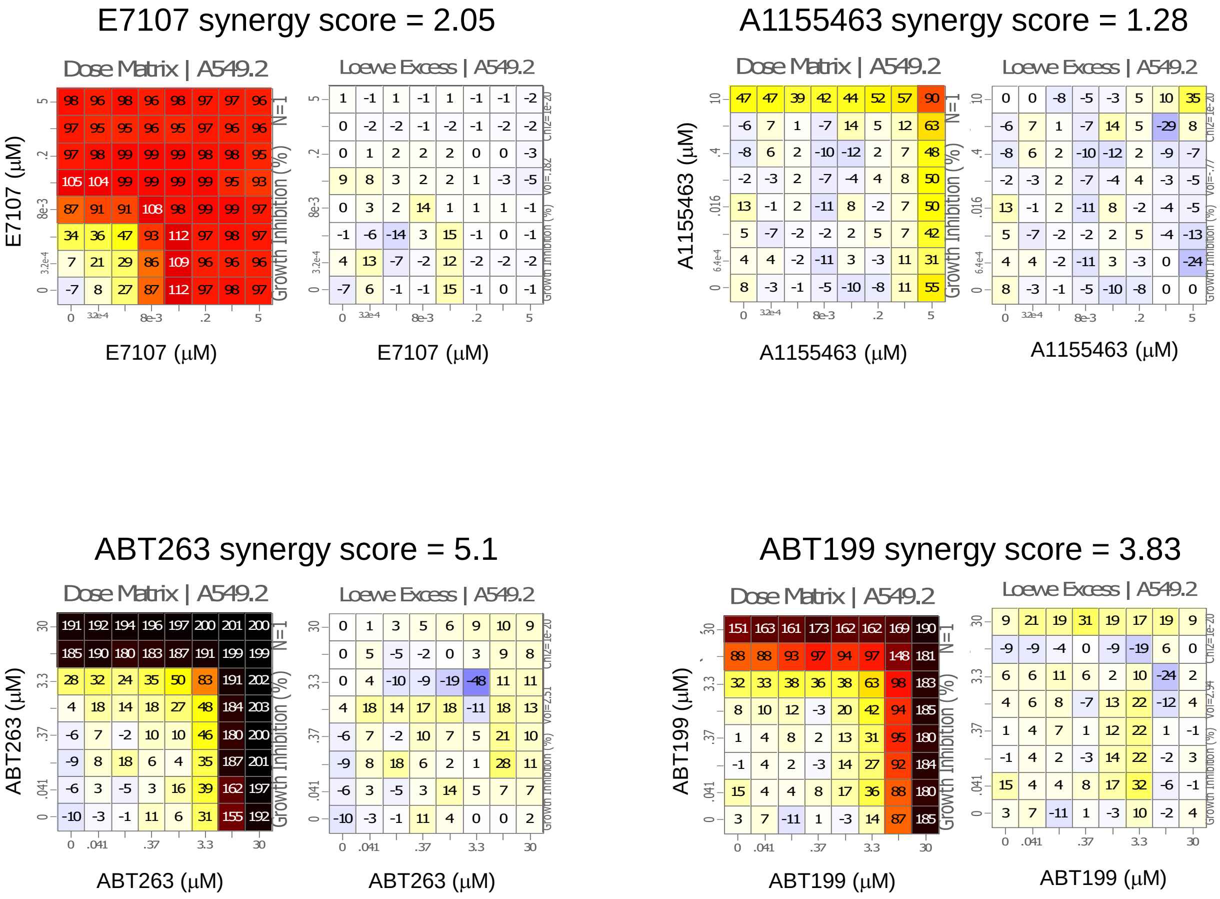

Supplementary Figure 11. Single agent "combination" does not show synergistic activity. 8x8 dose matrix single agent "combination" assays were conducted for E7107, A1155463, ABT263, and ABT199 in MCL1-independent NSCLC line A549. In the Loewe model, all of them showed synergy scores $\leq$ 5.

a

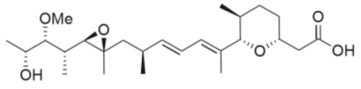

herboxidiene

b

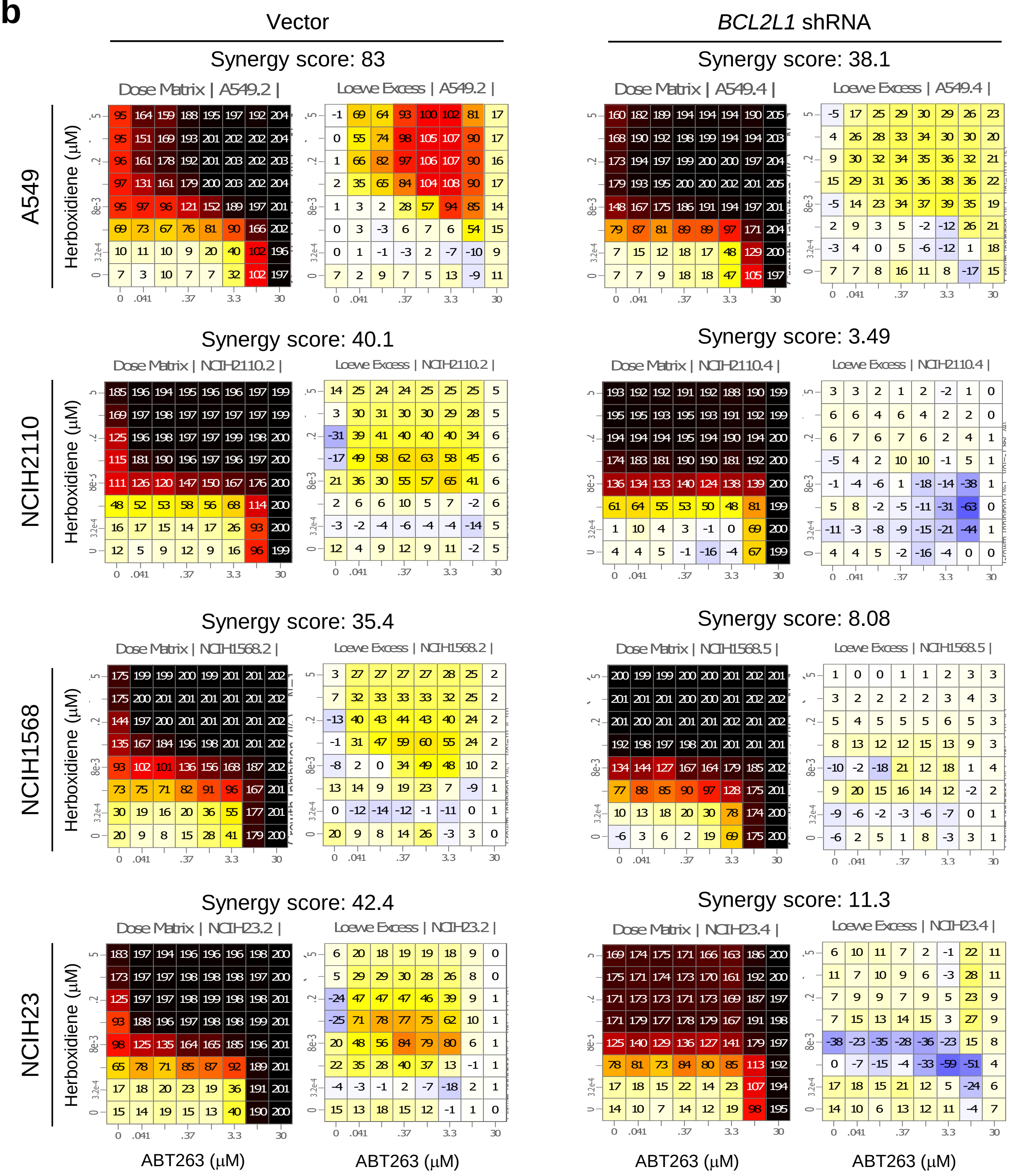

Supplementary Figure 12. Combination of herboxidiene and ABT263 induces synergistic cytotoxicity in NSCLC cell lines. (a) Chemical structure of SF3b-targeting splicing modulator herboxidiene. **(b)** 8x8 dose matrix combination study for herboxidiene derivative and ABT263 in MCL1-independent A549, MCL1-dependent NCIH2110, NCI1568 and NCIH23 cell lines stably transduced with vector control (Vector) or *BCL2L1* shRNA. Synergy scores were calculated by the Chalice software.

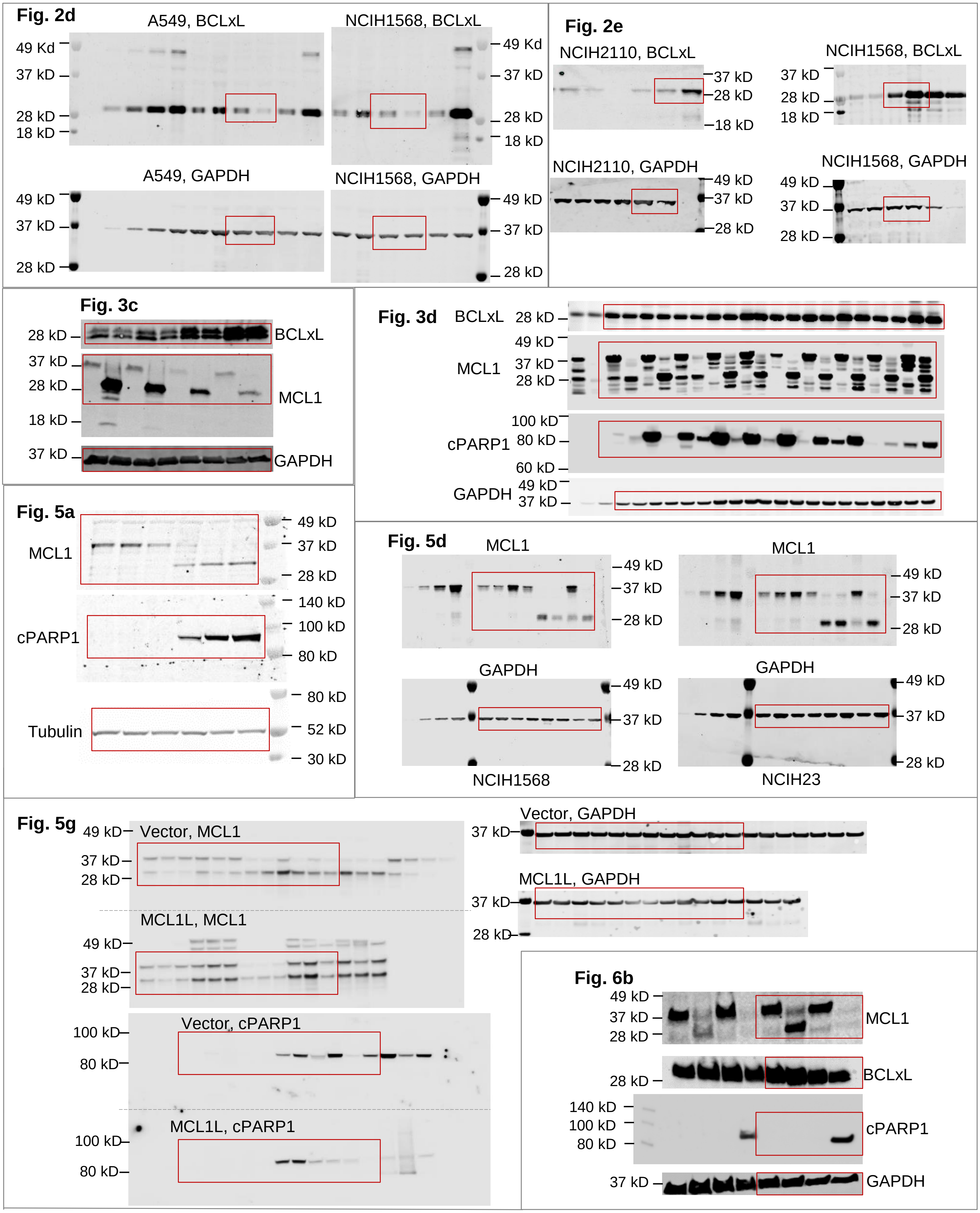

Supplementary Figure 13. Uncropped western blot images. Cropped region shown in indicated main figures are highlighted with red box.
