# Supplementary material for: Sensitivity to splicing modulation of BCL2 family genes defines cancer therapeutic strategies for splicing modulators
Source: Nat Commun. 2019 Jan 11;10:137. doi: 10.1038/s41467-018-08150-5 (PMC6329755; doi:10.1038/s41467-018-08150-5)
Supplement: Supplementary file 2 — Description of Additional Supplementary Files [file 41467_2018_8150_MOESM2_ESM.docx]

**Description of Additional Supplementary Files**

File Name: Supplementary Data 1

Description: 6.5K shRNA pool library (Excel file)

File Name: Supplementary Data 2

Description: Nalm6 E7107 shRNA screen data (Excel file)

File Name: Supplementary Data 3

Description: Cell line panel drug sensitivity (Excel file)

File Name: Supplementary Data 4

Description: Annotation of splicing variants of BCL2 family genes (Excel file)

File Name: Supplementary Data 5

Description: Mutation status of splicing genes in cell lines (Excel file)
