# Supplementary material for: Sensitivity to splicing modulation of BCL2 family genes defines cancer therapeutic strategies for splicing modulators
Source: Nat Commun. 2019 Jan 11;10:137. doi: 10.1038/s41467-018-08150-5 (PMC6329755; doi:10.1038/s41467-018-08150-5)
Supplement: Supplementary file 8 — Reporting Summary [file 41467_2018_8150_MOESM8_ESM.pdf]

nature research

Corresponding author(s): Ping Zhu

Reporting Summary

Nature Research wishes to improve the reproducibility of the work that we publish. This form provides structure for consistency and transparency in reporting. For further information on Nature Research policies, see Authors & Referees and the Editorial Policy Checklist.

Statistical parameters

When statistical analyses are reported, confirm that the following items are present in the relevant location (e.g. figure legend, table legend, main text, or Methods section).

n/a	Confirmed	
☐	☒	The exact sample size (n) for each experimental group/condition, given as a discrete number and unit of measurement
☐	☒	An indication of whether measurements were taken from distinct samples or whether the same sample was measured repeatedly
☐	☒	The statistical test(s) used AND whether they are one- or two-sided<br>*Only common tests should be described solely by name; describe more complex techniques in the Methods section.*
☐	☒	A description of all covariates tested
☐	☒	A description of any assumptions or corrections, such as tests of normality and adjustment for multiple comparisons
☐	☒	A full description of the statistics including central tendency (e.g. means) or other basic estimates (e.g. regression coefficient) AND variation (e.g. standard deviation) or associated estimates of uncertainty (e.g. confidence intervals)
☐	☒	For null hypothesis testing, the test statistic (e.g. F, t, r) with confidence intervals, effect sizes, degrees of freedom and P value noted<br>*Give P values as exact values whenever suitable.*
☒	☐	For Bayesian analysis, information on the choice of priors and Markov chain Monte Carlo settings
☒	☐	For hierarchical and complex designs, identification of the appropriate level for tests and full reporting of outcomes
☐	☒	Estimates of effect sizes (e.g. Cohen's d, Pearson's r), indicating how they were calculated
☐	☒	Clearly defined error bars<br>*State explicitly what error bars represent (e.g. SD, SE, CI)*

Our web collection on statistics for biologists may be useful.

Software and code

Policy information about availability of computer code

Data collection	We have specified all open source or commercial software to collect data in the Method section.
Data analysis	We have specified all open source or commercial software to analyze data in the Method section.

For manuscripts utilizing custom algorithms or software that are central to the research but not yet described in published literature, software must be made available to editors/reviewers upon request. We strongly encourage code deposition in a community repository (e.g. GitHub). See the Nature Research guidelines for submitting code & software for further information.

Data

Policy information about availability of data

All manuscripts must include a data availability statement. This statement should provide the following information, where applicable:

- Accession codes, unique identifiers, or web links for publicly available datasets
- A list of figures that have associated raw data
- A description of any restrictions on data availability

RNA-seq data have been deposited in NCBI's Gene Expression Omnibus (GEO) repository and are accessible through GEO Series accession numbers. The authors declare that all the other data supporting the findings of this study are available within the article and its supplementary information files and from the corresponding author upon reasonable request.

Field-specific reporting

Please select the best fit for your research. If you are not sure, read the appropriate sections before making your selection.

☒ Life sciences ☐ Behavioural & social sciences ☐ Ecological, evolutionary & environmental sciences

For a reference copy of the document with all sections, see nature.com/authors/policies/ReportingSummary-flat.pdf

Life sciences study design

All studies must disclose on these points even when the disclosure is negative.

Sample size	Determination of samples size and statistics are described in the method section.
Data exclusions	No data were excluded from the analyses in this study.
Replication	All replications were successful.
Randomization	Mice implanted with xenograft tumors were randomized at 150-200mm3 into experimental groups.
Blinding	In the animal studies, Investigators were blinded to the group allocation.

Reporting for specific materials, systems and methods

Materials & experimental systems

n/a	Involved in the study	
☒	☐	Unique biological materials
☐	☒	Antibodies
☐	☒	Eukaryotic cell lines
☒	☐	Palaeontology
☐	☒	Animals and other organisms
☒	☐	Human research participants

Methods

n/a	Involved in the study	
☒	☐	ChIP-seq
☒	☐	Flow cytometry
☒	☐	MRI-based neuroimaging

Antibodies

Antibodies used	MCL1 (Cell Signaling Technologies 5453) (D35A5) rabbit monoclonal diluted 1:500, BCLxL (Cell Signaling Technologies 2764) (54H6) rabbit monoclonal diluted 1:500, BCL2 (Cell Signaling Technologies D55G8) (4223) rabbit monoclonal diluted 1:500, BCL2L2/BCLw (R&D Systems AF824) rabbit polyclonal diluted 1:500, Cleaved Parp1 (Cell Signaling Technologies 5625) (D64P10) rabbit monoclonal diluted 1:500, Tubulin (Sigma T6199) mouse monoclonal at 50ng/mL, and GAPDH (Sigma G8795) mouse monoclonal at 100ng/mL. Anti-mouse IgG, HRP-linked Antibody (Cell Signaling Technologies 7076) and Anti-rabbit IgG, HRP-linked Antibody (Cell Signaling Technologies 7074) diluted 1:5,000 in blocking buffer.
Validation	Validation of all of the antibodies used was based on to manufacturer's validation experiments in the manuals or websites. In addition, anti-MCL1 and anti-BCLxL antibodies were validated by RNAi experiments from this study, and was shown in the manuscript.

Eukaryotic cell lines

Policy information about cell lines

Cell line source(s)	Cell lines were obtained from the American Type Culture Collection (ATCC) and cultured as per manufacturer's instruction.
Authentication	All cell lines have been tested for mycoplasma contamination and authenticated to confirm cell identity.
Mycoplasma contamination	All cell lines have been tested for mycoplasma contamination and authenticated to confirm cell identity.
Commonly misidentified lines (See ICLAC register)	None.

Animals and other organisms

Policy information about studies involving animals; ARRIVE guidelines recommended for reporting animal research

Laboratory animals	NCr nude female mice (6-8 weeks old) were sourced from Charles River (Wilmington, MA) and maintained in a pathogen-free facility according to Institutional Animal Care and Use Committee (IACUC) guidelines. All animal experiments were conducted according to IACUC guidelines defined by the H3 Biomedicine Animal Care and Use Program and study protocol.
Wild animals	No wild animals were used in this study.
Field-collected samples	No filed-collected samples were used in this study.
